# CVD Conditions for MWCNTs Production and Their Effects on the Optical and Electrical Properties of PPy/MWCNTs, PANI/MWCNTs Nanocomposites by In Situ Electropolymerization

**DOI:** 10.3390/polym13030351

**Published:** 2021-01-22

**Authors:** Silvia Beatriz Brachetti-Sibaja, Diana Palma-Ramírez, Aidé Minerva Torres-Huerta, Miguel Antonio Domínguez-Crespo, Héctor Javier Dorantes-Rosales, Adela Eugenia Rodríguez-Salazar, Esther Ramírez-Meneses

**Affiliations:** 1 Instituto Politécnico Nacional, CICATA-Altamira, Carretera Tampico-Puerto Industrial, km 14.5, Altamira 89600, Mexico; bbrachetti@hotmail.com; 2 Tecnológico Nacional de México, IT de Ciudad Madero, Ave Primero de Mayo S.N. Col. Los Mangos, Madero City 89449, Mexico; 3 Instituto Politécnico Nacional, CMPL, Av. Acueducto S/N, La Laguna Ticomán, México City 07340, Mexico; dianna_palma@hotmail.com; 4 Instituto Politécnico Nacional, UPIIH, Ciudad del Conocimiento y la Cultura, Carretera Pachuca-Actopan km. 1+500, San Agustín Tlaxiaca City 42162, Mexico; 5 Departamento de Metalurgia, Instituto Politécnico Nacional, ESIQIE, México City 07300, Mexico; hectordorantes@yahoo.com; 6 Instituto Politécnico Nacional, CICATA-Querétaro, Cerro Blanco 141, Col. Colinas del Cimatario, Santiago de Querétaro City 76090, Mexico; rodriguez_ade@yahoo.com; 7 Departamento de Ingeniería y Ciencias Químicas, Universidad Iberoamericana, Prolongación Paseo de la Reforma 880, Lomas de Santa Fe, México City 01219, Mexico; esther.ramirez@ibero.mx

**Keywords:** chemical vapor deposition, MWCNTs, conducting polymers, hybrid composites, stability, electrical properties

## Abstract

In this work, the optimal conditions of synthesizing and purifying carbon nanotubes (CNTs) from ferrocene were selected at the first stage, where decomposition time, argon fluxes, precursor amounts, decomposition temperature (at 1023 K and 1123 K), and purification process (HNO_3_ + H_2_SO_4_ or HCl + H_2_O_2_), were modulated through chemical vapor deposition (CVD) and compared to commercial CNTs. The processing temperature at 1123 K and the treatment with HCl + H_2_O_2_ were key parameters influencing the purity, crystallinity, stability, and optical/electrical properties of bamboo-like morphology CNTs. Selected multiwalled CNTs (MWCNTs), from 1 to 20 wt%, were electropolymerized through in-situ polarization with conductive polymers (CPs), poly(aniline) (PANI) and poly(pyrrole) (PPy), for obtaining composites. In terms of structural stability and electrical properties, MWCNTs obtained by CVD were found to be better than commercial ones for producing CPs composites. The CNTs addition in both polymeric matrixes was of 6.5 wt%. In both systems, crystallinity degree, related to the alignment of PC chains on MWCNTs surface, was improved. Electrical conductivity, in terms of the carrier density and mobility, was adequately enhanced with CVD CNTs, which were even better than the evaluated commercial CNTs. The findings of this study demonstrate that synergistic effects among the hydrogen bonds, stability, and conductivity are better in PANI/MWCNTs than in PPy/MWCNTs composites, which open a promissory route to prepare materials for different technological applications.

## 1. Introduction

Conductive polymers (CPs) and conductive polymer composites (CPCs) are promising semiconductor materials for a wide variety of applications because of their tunable physical/chemical properties, mechanical flexibility, low weight, reversible doping, adequate bio-compatibility, and large-scale production [1]. Different conjugated polymers such as polythiophene [2,3], polypyrrole (PPy) [4,5], poly(3,ethylenendioxythiophene) (PEDOT) [6,7], and polyaniline (PANI) [8,9], have been explored as engineering polymers (multifunctional materials). Among the CPs, PPy and PANI are considered favorable materials for multiple applications, such as capacitors, redox capacitors, lithium-ion batteries, super-condensers, filtering membranes, water treatment, and biosensors [10,11,12,13,14,15,16,17,18,19].

Additionally, PANI and PPy nanocomposites with different embedded nanostructures combined the synergistic effect of each one to obtain singular properties. These conjugated polymers are reinforced with either carbon nanotubes (CNTs), reduced graphene oxide powders, or metallic or ceramic nanoparticles, promoting the overall electrical conductivity in a wide variety of applications [20,21,22,23,24,25]. Even small concentrations of these nanostructures can promote the formation of percolating networks, improving electrical properties [26]. Several factors have an effect on the composite electrical conductivity including characteristics of the polymer, production process, and type and fraction of filler. For example, PANI can present different oxidation degrees, since fully oxidized (per)nigraniline, partially oxidized emeraldine and fully reduced leucomeraldine, modify its electrical conductivity [3]. A conductivity between 32 and 223 S cm^−1^ has been reported as a function of CNTs content [27]; whereas PANI has shown values between 0.5 to 100 S cm^−1^ [28,29]. Thus, whereas some researchers are focused on the purity and construction of the fillers, other efforts are being realized to determine the optimal ratio polymer/filler to increase the electrical conductivity through a large electrochemical surface area [30]. Among the carbon materials, multi-walled CNTs (MWCNTs) present an excellent electrical conductivity with unique structure, adequate mechanical properties, and large effective surface area, which help to improve the composite stability [31]. Recent reports of PPy/CNTs and PANI/CNTs nanocomposites are related to the effects of the CNTs incorporation into the CPs and the morphological aspects (interfacial area, aspect ratio, and interfacial adhesion); however, those effects on the electrical conductivity are not fully understood. On the other hand, the most common methods for the synthesis of MWCNTs are arc discharge, laser ablation, and chemical vapor deposition (CVD) [32,33]; although other methods have also been proposed, such as arc decomposition of SiC, torsion of graphene layers, heat treatment of polymers, and pyrolysis electrolysis [34,35,36,37]. CVD is either thermal or plasma enhanced, and it has become a standard method for the CNTs production, as it is considered a large scale and high purity method. Unfortunately, the control parameters and CNTs growth are still under discussion; due to the characteristics of carbon nanotubes depending on the working conditions [38,39,40]. For example, the yield and quality of CNTs are greatly affected by the matrix material, and they are conditioned by their morphology and alignment. Thus, for several applications such as micro-nanoelectronics devices, sensors, electric actuators, and optical devices, among others, it is essential to optimize CVD conditions.

In this paper, a systematic study is reported to evaluate the different parameters that influence the stability and electrical conductivity of CPs (PPy or PANI)/MWCNTs nanocomposite films to be potentially used as sensors or thermoelectric devices. During the first set of experiments, it was realized the synthesis of MWCNTs by thermal chemical vapor decomposition (CVD) at different experimental conditions. These samples were acid functionalized and characterized to select the optimal conditions to produce nanocomposites, from high purity MWCNTs and conjugated polymers, i.e., PPy or PANI, using polymerization assisted through in-situ polarization technique (potentiostatic method). The synergistic electronic interaction has been analyzed as a function of different amounts of MWCNTs (0–20 wt%) in both CPs. A special focus has been placed on the structure, stability, and electrical conductivity of the CPs (PPy or PANI)/MWCNTs nanocomposites.

## 2. Experimental Procedure

### 2.1. Synthesis and Purification of Carbon Nanotubes (CNT)

MWCNTs powders were obtained by a hot-wall CVD process at atmospheric pressure, using ferrocene (C_10_H_10_Fe, Sigma-Aldrich, Mexico City, Mexico, 98%) as single source precursor and catalyst. The CVD experimental setup consisted of a three-zone horizontal furnace (HTF55347C model, Lindberg/blue M) with a tubular quartz reactor (120 cm in length and 5 cm in diameter), under argon (F, flow rate of carrier gas: 50–100 sccm) atmosphere. The initial weight of C_10_H_10_Fe (W) was of 0.1 and 0.2 g, which were placed into the equipment using a precursor temperature (T_prec_) of 473 K, temperature previously obtained from thermogravimetric analysis, whereas the vapors were transported into the hot zone at two decomposition temperatures (T) of 1023 and 1123 K, and two decomposition times (t) of 30 and 60 min.

Purification of CNTs powders fabricated at different experimental conditions was done with an acid solution composed by 20 mL of 3 M hydrochloric acid (HCl, Sigma-Aldrich, Mexico City, Mexico, 37%) + 20 mL of a solution 30 vol.% of hydrogen peroxide (H_2_O_2_, Sigma-Aldrich, Mexico City, Mexico, 30–50%). Then, 60 mg of powders was added into the purified solution and it was maintained under stirring at 348 K for 4 h under reflux conditions. Thereafter, dispersion was neutralized with deionized water and ethanol, subsequently centrifuged at 2000 rpm during 5 min, and filtered and dried at 373 K for 24 h.

To observe the influence of the chemical attack on the CNT structure, samples synthesized at 1123 K were purified with a different procedure. A mixture of 20 mL of nitric acid 3 M (HNO_3_, Sigma-Aldrich, Mexico City, Mexico, 70%) + 20 mL of sulfuric acid 30 vol.% (H_2_SO_4_, Sigma-Aldrich, Mexico City, Mexico, 95–98%) was prepared. Subsequently, 60 mg of obtained sample was processed using a similar procedure.

Additionally, commercial multi-walled CNTs, (product name: carbon nanotube, multi-walled, product number: 791431, Sigma-Aldrich, Mexico City, Mexico, 70–80% pure, 10 × 4.5 × 4 mm, surface resistivity 700–900 Ω square^−1^ by 4-point probe method) were also purified by both methods and characterized for comparison.

### 2.2. Fabrication of Polymer/MWCNTs Nanocomposites

Two conducting polymers were synthesized using a potentiostatic method. Electrolytic and oxidant agents as well as the monomer used to synthesize poly(pyrrole) were oxalic acid (C_2_H_2_O_4_, Sigma-Aldrich, Mexico City, Mexico, 98%), lithium perchlorate (LiClO_4_, Sigma-Aldrich, Mexico City, Mexico, 98%) and pyrrole (C_4_H_5_N, Sigma-Aldrich, Mexico City, Mexico, 98%), respectively. In the case of poly(aniline), HCl, ammonium persulfate ((NH_4_)_2_S_2_O_8_, Sigma-Aldrich, Mexico City, Mexico, ≥98%) and aniline (C_6_H_5_NH_2_, Sigma-Aldrich, Mexico City, Mexico, ≥99%) were used. Based on previous reports, the following procedure was realized [41]: either the PPy/MWCNTs or PANI/MWCNTs composite films were electrodeposited on commercial AISI 1018 carbon steel (35 × 75 mm) or AISI 316L stainless steel(35 × 75 mm), respectively. As counter electrode, an AISI 316L stainless steel plate with the same area was used.

Different amounts of MWCNTs (1, 5, 6.5, 8, 10, and 20 wt%) were prepared with a solution of 0.1 M oxalic acid + 0.1 M LiClO_4_ in a 1:1 ratio (*v*/*v*); thereafter, 50 mL of this solution was mixed with 50 mL of a pyrrole solution (0.3 M) and sonicated for 30 min. Then, 200 mL of this dispersion as placed into the cell compartment, in which the 316L stainless steel and AISI 1018 carbon steel were working and counter electrodes, respectively. The nanocomposites were obtained in situ by applying a constant potential of −1100 mV_Eocp_ vs. SCE for 30 min and then kept under mechanical stirring again for 30 min.

For PANI nanocomposite films, a potential of −600 mV_Eocp_ was applied vs. SCE for 30 min, but in this case, to assurance emeraldine formation, a solution of 0.1 M HCl + 0.1 M (NH_4_)_2_S_2_O_8_ in a 1:1 ratio (*v*/*v*) was prepared. This solution was added into 0.3 M of aniline following the same procedure with the exception that the system in the electrolytic cell was kept at 383 K in a cooling bath while using the 316L stainless steel as working and counter electrodes. A power supply (BK Precision 1746B) was used in the potentiostatic experiments.

### 2.3. MWCNTs and Nanocomposites Characterization

To verify the structure and purity of MWCNTs, X-ray-diffraction (XRD) patterns were realized in a D8 Advance, Bruker diffractometer with a Cu K*α* radiation (*λ* = 0.15405 nm). The equipment was operated at 35 kV and 25 mA in a *θ*-2*θ* configuration. The evaluated range was from 2*θ* = 20° to 70° at a speed of 0.02° min^−1^.

Ultraviolet-visible (UV-vis) and Raman spectroscopies were also used to study the molecular structures of pure compounds (MWCNTs and CPs), as well as the interfacial structure between both components forming the composites [42]. UV-vis spectra were acquired in aqueous solution (0.1 mg·mL^−1^); previously the samples in powders form were dispersed into deionized water, using a Cary 5000 UV-vis-NIR spectrometer (Agilent Technologies) in the 200–1000 nm range with a scan rate of 600 nm min^−1^.

Raman studies were realized in a Renishaw InVia microscope using an excitation source of 514 nm and a laser power of 20 mW. The samples were scanned from 500–3000 cm^−1^ shift at spatial resolution of 2 cm^−1^. The scanning parameters for the spectra were: 10 s of acquisition, an objective of 100×, and a numerical aperture of 0.85. Five scans were acquired for each experiment to assurance and adequate signal to noise ratio.

Fourier transform infrared (FT-IR) was collected in the range from 4000 cm^−1^ to 450 cm^−1^ using a Perkin–Elmer Spectrum One 51,394 series spectrometer equipped with an attenuated total reflectance accessory (ATR). Thermogravimetric analysis was realized in a Labsys Evo, Setaram TGA/DSC equipment. Then, 10 mg of each sample was placed in alumina crucibles and heated from 298 K to 1123 K using a heating rate of 10 K min^−1^ under argon atmosphere (flow rate: 20 mL min^−1^). Samples were maintained for 5 min at 1123 K and then cooled up to room temperature.

The morphological aspects of the MWCNTs and composites were analyzed by scanning electron microscopy using a JEOL JSM-6701F microscope at a 5 kV acceleration voltage. Samples were sputter coated with Au-Pd for 30 s on a Quorum Q150T ES sputter coater system. Electrical measurements of both kinds of nanocomposites were performed by a conventional 4-point collinear probe using a Keithley Model 2410C instrument. The analysis was realized using a software based on the proportional integral-derivative approach (PID). For these measurements, composite powders obtained were compressed at 528 MPa to get pellets (10 mm in diameter and 1.7 mm in thickness) followed by a sintering process at 373 K for 4 h. Reported electrical conductivity values were the average of three samples for each condition. The Hall Effect measurements were realized using van der Pauw configuration at room temperature using an ECOPIA HMS 3000 apparatus.

## 3. Results and Discussion

### 3.1. Characterization of MWCNTs before and after Purification Process

The synthesis of CNTs using hydrocarbon sources as CH_4_, C_2_H_2_, EtOH, or CO is expensive because of the complex purification process [43], whereas the used of ferrocene at atmospheric pressure can be an alternative to obtain CNTs with adequate carbon yield; then, an optimization of CVD parameters in one step is essential to reduce the costs production in comparison with other reported methods [44]. Among different carbon sources used as raw materials (ferrocene, graphite, methane, and coal derived hydrocarbons), coal is cheaper and an abundant carbon source. Unfortunately, the cost of different carbon source as raw material, for CNTs production, is not the determining factor in the final production cost [45]. For example, the yield and purification process can be more important that raw materials costs [46].Then, during the synthesis, CNTs can contain a large amount of impurities such as amorphous carbon, multishell carbon nanocapsules, or even metal particles, depending of the carbon source. In fact, the use of alcohols as feedstock produces high purity carbon nanotube synthesis at low temperature synthesis [47], but other raw materials, such as ferrocene, can also be an option.

**Figure 1:**
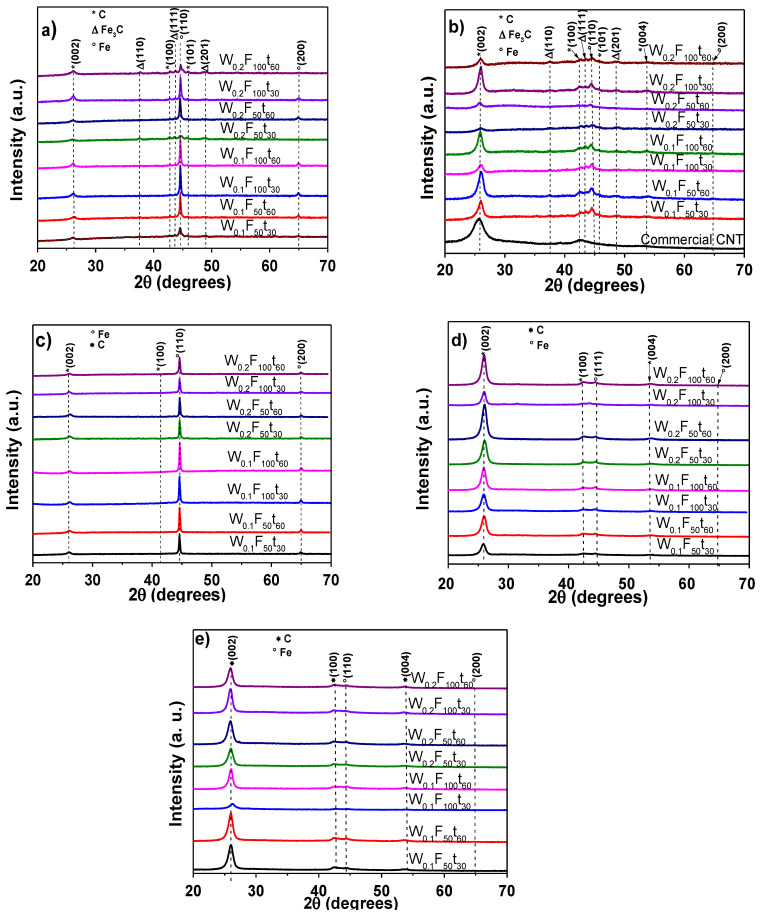
XRD patterns of MWCNTs obtained with different synthesis parameters at 1023 K (**a**) unpurified samples, (**b**) purified samples with HCl + H_2_O_2_ solution. Samples synthesized at 1123 K (**c**) unpurified samples, (**d**) purified samples with HCl + H_2_O_2_ solution, and (**e**) purified samples with HNO_3_ + H_2_SO_4_.

Figure 1a–d shows the XRD results for MWCNTs obtained at 1023 and 1123 K, using different CVD parameters: decomposition time of 30 and 60 min (t), argon fluxes at 50 and 100 sccm (F), and two different amounts of precursors (W, 0.10 and 0.20 g). Particularly, Figure 1a shows XRD patterns before chemical purification, whereas Figure 1b shows the structural changes in relation to each condition after purification (1023 K). From Figure 1a, it can be seen the main signals of Fe at 45.86° (110) and 64.76° (200), PDF 06-0696 chart. Additionally, small signals at 37.50° (110), 43.40° (111), and 48.40° (201) matched well with the Fe_3_C in the structural BCC phase (PDF 00-003-0400). MWCNTs pattern shows weak reflections at 26.10° (002), 42.80° (100), and 45.80° (101), according to the PDF 00-002-0456 chart. As expected, the purification of MWCNTs reduces the signals of the Fe and Fe_3_C structural phases (Figure 1b). In fact, Fe_3_C reflections almost disappear, whereas MWCNTs reflections become stronger. The main XRD peaks of MWCNTs matched well with the commercial MWCNTs under similar purification process. The effect of the CVD temperature (1123 K) with and without purification is shown in the Figure 1c,d.

For all the samples, the XRD patterns were very similar, but an increase of 373 K was enough to eliminate the Fe_3_C phase formation. The acid treatment with HCl almost removed the amorphous carbon structures and iron reflections; a small deviation of the (002) peak of the purified samples can be related with an increase in the carbon inter-layer spacing [48]. The effect of the purification process of CNTs on the electrical properties has been widely reported [49,50], even though an adequate purification degree contributes to improve electrical properties of carbon nanotube-polymer composites; however, it still remains a challenge [51]. Differences in the electrical conductivity are commonly related to type and quantity of CNTs, purity, different sizes, crystallinity and orientation, straightness, and entanglement [52]. The CNTs samples without Fe_3_C phase were purified with a different chemical treatment (HNO_3_ + H_2_SO_4_) to observe changes in the crystallinity degree. XRD pattern of purified sample with this solution is shown in Figure 1e. No clear trend can be observed in the XRD patterns, but it is similar to that with purified samples with HCl + H_2_O_2_ solution, the signals of iron impurities almost disappeared.

To analyze possible changes in the crystallite size, an estimation of overall samples was realized from typical Scherrer equation $t=\;\frac{0.9\lambda }{\beta \;\cos\theta }$; where *t* is crystallite size, *λ* = CuK*α* wavelength (0.15405 nm), *β* = FWHM (full width at half maximum), and *θ* = Bragg angle. Additionally, the crystallinity degree of MWCNTs was calculated from a two-phase model (Equation (1)) in combination with a deconvolution process (pseudo-Voigt peak function) [53].

(1) $$W_{c,x}=\;\frac{\sum_{i}C_{i,\;hkl}\left(\theta \right)\;I_{i,\;hkl}\left(\theta \right)}{\sum_{i}C_{i,\;hkl}\left(\theta \right)\;I_{i,\;hkl}\left(\theta \right)+\;\sum_{j}C_{j}\left(\theta \right)I_{j}\left(\theta \right)k_{i}}\;\times 100\%$$

In this equation, $W_{c,x}$ is the percent crystallinity; *i*, *j*, are the numbers of crystalline diffraction peaks from MWCNTs and amorphous scattering peaks from amorphous carbon, respectively. $C_{i,\;hkl}\left(\theta \right),\;I_{i,\;hkl}\left(\theta \right)$ are the correction factor and integral intensity of the crystalline diffraction peak, respectively; $C_{j}\left(\theta \right),\;I_{j}\left(\theta \right)$ are the correction factor and integral intensity of the amorphous scattering peak; $k_{i}$ is the correction coefficient, in this case, $k_{i}$ = 1 (Table 1).

The comparison of both chemical treatments indicated that crystallite size is reduced during the purification process. On the other hand, the percent crystallinity is higher in samples treated with HCl + H_2_O_2_, i.e., the chemical attack in the carbon structures is more severe in the produced MWCNTs using an oxidation with a HNO_3_ + H_2_SO_4_ solution. It has been reported that an oxidation of CNTs with HNO_3_ solution is more efficient in reducing the amorphous carbon phase as well as metal impurities in the CNTs than other oxidants; however, it can also affect the attack of the carbon structure breaking the CNTs into shorter segments [54,55]. Furthermore, other researches have referred that nitric acid can attack the preferably defective sites and intercalate into the CNTs to unzip the tube walls by further oxidative etching. This provokes an increase of the tube interlayers again while reducing the crystallinity of the samples [56]. The above results led to discriminate the samples according to the purity and percent crystallinity. Therefore, only the MWCNTs samples obtained at 1123 K and purified with an acid solution of HCl + H_2_O_2_ were used for further characterization and used to produce MWCNTs/polymer composites.

**Figure 2:**
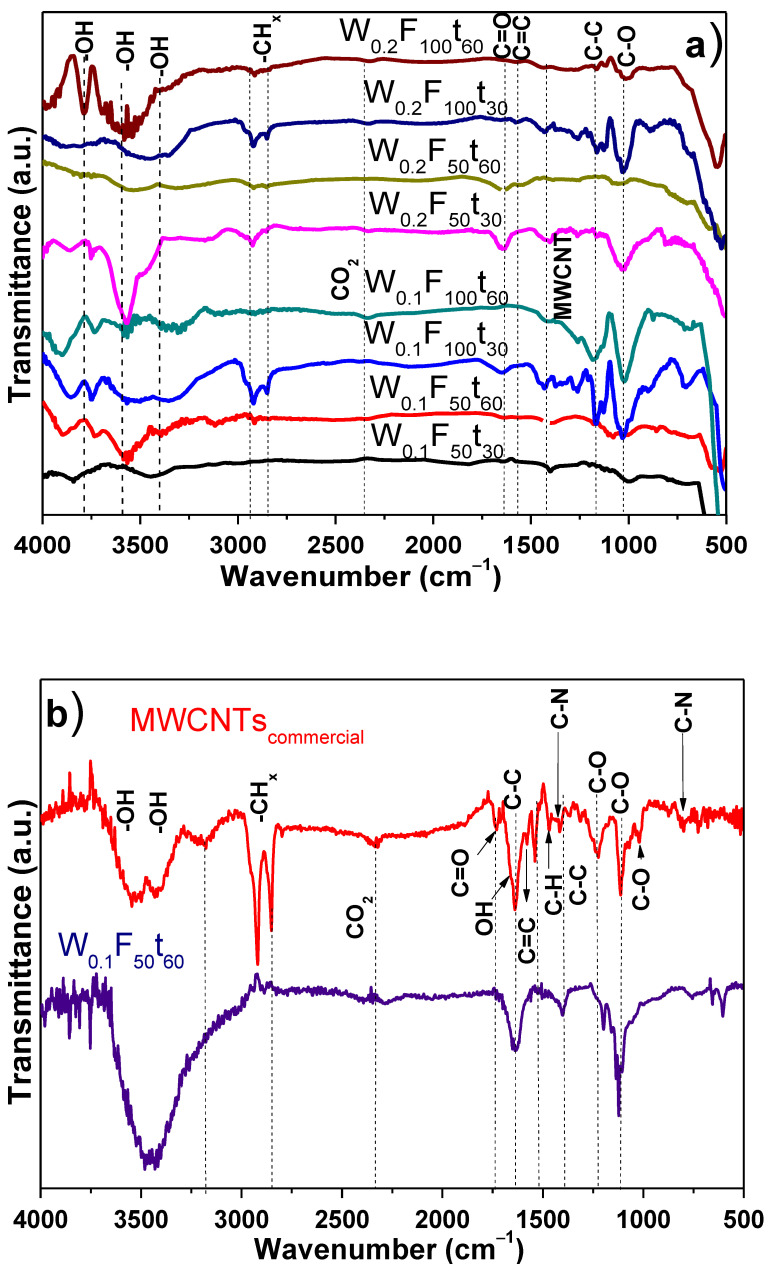
FT-IR spectra from MWCNTs at 850 °C, after purification with 3 M HCl + 30 vol.% H_2_O_2_: (**a**) samples at different conditions; (**b**) comparison between commercial MWCNTs and a selected sample.

FT-IR was also used to examine the MWCNTs functionalization and to compare it with commercial MWCNTs under similar purification process (Figure 2a,b). From these figures, the following bands were identified in the synthesized MWCNTs: –OH stretching from unbound or free hydroxyl of phenol between 3800 and 3700 cm^−1^, –OH bands among 3300 and 3700 cm^−1^, and asymmetric and symmetric stretching of CH_x_ groups are observed between 2900–2800 cm^−1^. The band in the wavenumber of 2330 cm^−1^ is associated with the CO_2_ from the air. The stretching of C≡C bonds is slightly observed at 2140 cm^−1^, whereas that of C=C bonds is at 1560 cm^−1^. The peak at 1660cm^−1^ is correlated with the C=O stretching presence of the carboxylic acid group (–COOH). Vibrational modes associated with MWCNTs are observed at 1445 and 1575 cm^−1^, C–C bands must be located at 1377, and C–O bonds of the carboxyl group are obtained at 1010 cm^−1^ [57,58,59,60].

On the other hand, commercial MWCNTs showed the typical O–H bands in the region from 3300 to 3700 cm^−1^ and C–H stretching bands at 3180 and 2800 cm^−1^ [61,62], respectively. Additionally, the CO_2_ trapped into the MWCNTs was observed at 2330 cm^−1^ [63,64]. The OH groups of adsorbed water as well as to the covalently bonded functional groups are observed at 1650 cm^−1^ [65]; C=C bonds in aromatic ring at 1580 cm^−1^; and rocking and deformation out of plane vibrations of CH_2_ or CH_3_ groups at 1467 cm^−1^. Similarly, 1414 and 800 cm^−1^ are the stretching of C–N groups. The bands at 1228 cm^−1^, 1110 cm^−1^, and 1020 cm^−1^ correspond to the C–O stretching/OH bending vibrations and/or the stretching of C–O in ester, ether, and phenol of carboxyl, respectively [66,67]. The most important differences between synthesized and commercial MWCNTs are: (i) the OH groups from unbound or free hydroxyl of phenol are missing in the commercial samples, (ii) the CH_2_ or CH_3_ groups only appear in commercial samples, and (iii) there is C–O shifting due to the chemical environment in the synthesized CNTs. In all synthesized CNTs, there is the presence of two bands at 605, 757, and 655 cm^−1^ that arise after purification with HCl and H_2_O_2_. Because the synthesized MWCNTs can present some structural imperfections, there are some carbonaceous materials (such as graphitic carbons, carbon nanoparticles and amorphous carbon coatings) that might contribute to the appearance of these bands [40]. The identification of vibrational modes (1445 and 1575 cm^−1^), attributed uniquely to MWCNTs, leads to a better interpretation of FT-IR results [68,69]. Then, even synthesized MWCNTs present some differences, the appearance of OH, –COOH, and –CO groups is typical of an adequate MWCNTs functionalization.

The structural dispersion and stability of CNTs have been correlated with the absorption intensity. That is, better dispersion-stability, greater absorption intensity, and minimum changes in the spectrum should appear [70,71,72]. For this reason, the samples were evaluated through the UV-vis analysis. The UV-vis technique gives the MWCNTs spectrum according to their diameters and chiralities [70,73]. Additionally, it helps to identify single-walled CNTs (SWCNTs) and multiwalled CNTs (MWCNTs) depending on the absorption peak at specific wavelength, i.e., MWCNTs are related to the absorption at around 250–275 nm [70,71,74], whereas SWCNT present an absorption at 972 nm and 1710 nm [70,75].

**Figure 3:**
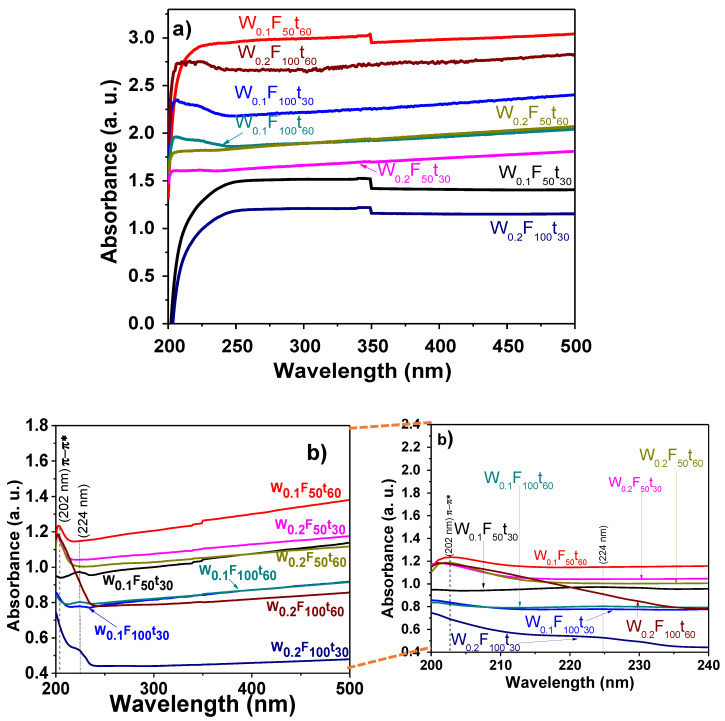
UV-Vis spectra of MWCNTs synthesized at 1123 K with different precursor amounts, reaction times, and argon flow rates: (**a**) before purification; (**b**) after purification with 3 M HCl + 30 vol.% H_2_O_2_ with a magnification between 200–240 nm.

Figure 3 shows the UV-vis spectra of aqueous suspended CNTs, before (Figure 3a) and after (Figure 3b) purification with 3 M HCl + 30 vol.% H_2_O_2_. The UV-vis of commercial carbon nanotubes is presented as supplementary material (Figure S1 in the Supplementary Material). The spectra of MWCNTs before purification do not display any significant peak in the region between 200 and 500 nm, mainly because of the presence of some structures (as amorphous and graphitic carbon) and the hydrophobic nature of CNTs. There is only the presence of a weak absorption maximum detected at 206 nm, which is only evident in samples with the highest used content of ferrocene to produce the MWCNTs; this band relates to *π*-plasmon resonance [76]. With respect to the absorbance values, a clear tendency with the weight of ferrocene used and the flux was not found; the only feature observed is that the CNTs produced for 60 min absorb more than their corresponding samples, those for 30 min. The results can be explained in terms of the wettability (hydrophobic/hydrophilic nature) of the as-prepared CNTs, which can modify the optical absorption properties (transparency) [77]. In agreement with previous research, in this study, the wettability of CNTs modified UV-Vis measurements due to their arrangement (orientation) and/or roughness [78].

Commercial CNTs typically show the characteristic band at 272 nm, attributed to the *π*-plasmon absorption [79], and the band at 222 nm corresponds to the collective excitations of the *π*-band electrons (common band feature in CNTs at ~175–245 nm) [80,81]. In this study, these bands appear at 202 and 224 nm (inset Figure 3b). The samples with a high intensity were W_0.1_F_50_t_60_, W_0.2_F_50_t_30_, W_0.2_F_50_t_60_ (202 nm) and W_0.1_F_50_t_30_, W_0.1_F_100_t_60_, and W_0.2_F_100_t_30_ (224 nm); these bands appear by the *π*-plasmon resonance, common in CNTs [80,81]. No clear trend in the absorbance values was observed in relation to the processing parameters; however, all the samples show a reduction in the absorbance values after the purification process.

**Figure 4:**
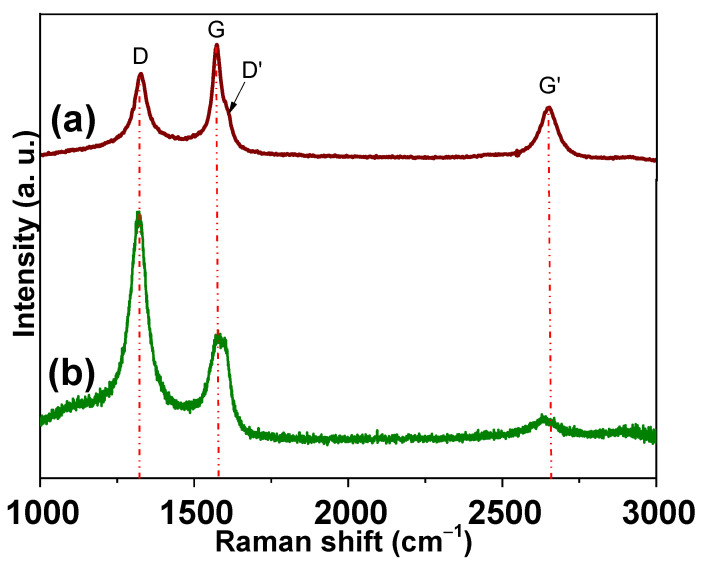
Raman spectra acquired of (**a**) Selected MWCNTs synthesized at W_0.1_F_50_t_60_T_1123_ followed with an acid purification (HCl + H_2_O_2_) and (**b**) commercial MWCNTs purified under similar conditions.

Another important feature to analyze in the MWCNTs is evaluated structural changes after purification; for this reason, a selected sample synthesized with the following parameters W_0.1_F_50_t_60_ and purified with acid chemical treatment (HCl + H_2_O_2_) was analyzed by Raman spectroscopy (Figure 4a). In this figure, the Raman shift of the commercial MWCNTs under similar conditions of purification is also shown (Figure 4b). This technique is widely used to analyze carbon materials, and it provides significant data about the carbon structures [82]. Three typical bands are identified in both spectra; however, a slight displacement of Raman shifting in the synthesized CNTs at 1326 (D), 1574 (G), and 2649 (G′) cm^−1^ is observed in comparison with commercial CNTs appearing at 1318 (D), 1581(G), and 2641(G′) cm^−1^ [83]. Additionally, in Figure 4a, a slight shoulder near to G band around 1608 cm^−1^ is observed (D′). This band was narrower than in not purified CNTs, and it is evidence of an adequate purification process. D′ is also related to the lattice vibration of defective graphite-like materials [84,85]. In the case of CNTs structures, the D band (disorder band) is identified as the A_1g_ symmetrical stretching coming from disorder and defects present in CNTs [86]; the G band (graphitic band) is associated with tangential phonon modes; the G′ band is an overtone of the D band, which is related to the stacking order of graphene sheets [87]. The amount of defects of the synthesized is about 4% of the curve, confirming that the synthesis and purification process were adequate [88]. Meanwhile, the I_G’_/I_G_ ratio also confirms that the structural defects are higher in the as-obtained sample (0.55) in comparison with commercial CNTs (0.21). The I_D_/I_G_ ratio has been widely used to evaluate the structure’s order; thus, a value near zero is desirable [89]. The I_D_/I_G_ and I_G’_/I_G_ ratios were obtained using the maximum heights of the D, G, and G’ bands after subtracting a baseline. As-prepared CNTs (W_0.1_F_50_t_60_) show an I_D_/I_G_ ratio of 0.81 versus 2.11 for commercial CNTs. These results indicate that commercial CNTs display less order structures than the selected sample.

**Figure 5:**
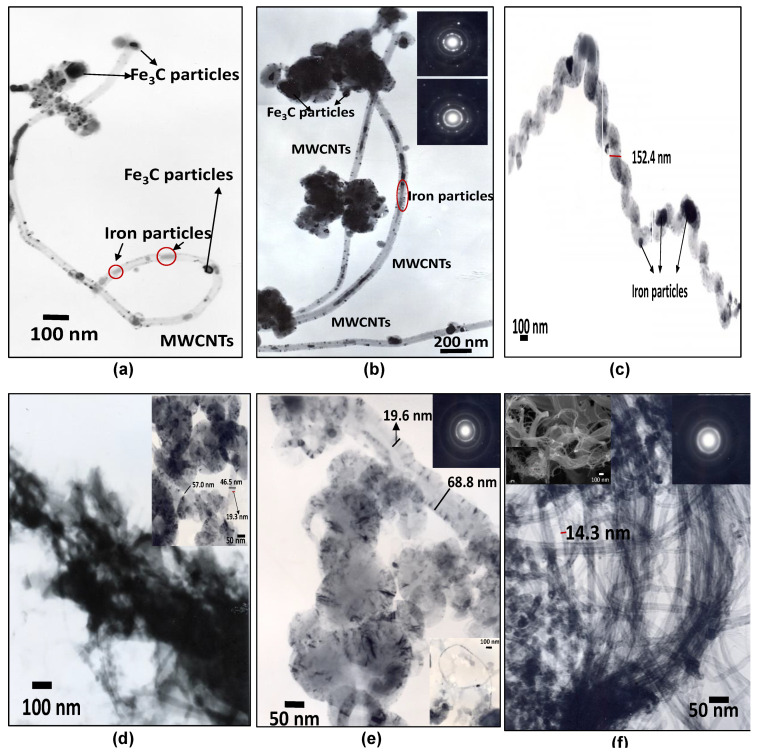
Selected TEM images of MWCNTs, (**a**–**c**) W_0.1_F_50_t_60_T_1123_ before purification, (**d**,**e**) W_0.1_F_50_t_60_T_1123_ after purification with HCl:H_2_O_2_ (**f**) commercial MWCNTs after purification with HCl:H_2_O_2_.

TEM micrographs and selected area electron diffraction (SAED) patterns were used to confirm structure and morphological features before and after purification (Figure 5a–f); the comparison with commercial MWCNTs is also presented in this figure. TEM micrographs show that during the CNTs synthesis by CVD process, some dispersed iron particles are trapped inside CNTs walls, whereas other particles of a combination of Fe and FeC are located over the walls surface (Figure 5a,b). Both types of particles come from the ferrocene precursor used during the synthesis of MWCNTs, and the nanostructure nature is shown in the inset SAED patterns. The synthesized MWCNTs show a bamboo-like morphology [90], combined with a helical structure from coiled carbon tubes linked to an iron particle (Figure 5c) [91].

The coiled tubes present “elbow-like junctions”, making it difficult to measure the diameter, but the walls of these CNTs are thick [48,92,93]. After purification (Figure 5d), the CNTs appear as aggregates, perhaps due to the absence of graphitic edges [94]. A magnification of the aggregates shows the helical MWCNTs structures, and it still presents a small quantity of Fe nanostructures, eliminating the FeC, which confirms XRD results. The external diameter was found between 46.5 and 68.8 nm with a wall thickness from 19.3 to 19.6 nm. The coils forming the helical structure implicate the presence of defects that are responsible for the D’ band observed previously in Raman spectra. The quantity of iron that remains after purification process suggested a tip growth mechanism; in this mode of growth, catalyst particle places at the top of CNTs, and it rises with the development of CNTs, capturing and encapsulating, sometimes, the particle during the growth process [95,96]. The above characterizations indicate that MWCNTs grow by the dissolving–diffusion–precipitation model, which is explained elsewhere [90,95]. TEM micrographs of commercial MWCNTs after purification are shown in Figure 5f; for comparison, these micrographs show a more similar morphology than that presented by as-synthesized samples, although the external diameter is smaller ~14.3 nm.

### 3.2. Chemical Analysis of Electrochemical PPy/MWCNTs, PANI/MWCNTs Composites

Electropolymerization of samples was carried out using a potentiostatic method in two different solutions to obtain PPy/MWCNTs and PANI/MWCNTs film composites. Different amounts of the synthesized and commercial MWCNTs powders were added in the electrolyte at 1, 5, 6.5, 8, 10, and 20 wt% on the growth film to evaluate the effect on the electrical properties. Additionally, diverse overpotentials were analyzed until they reached an adequate potential for PPy of −1100 mV_Eocp_ vs. SCE and PANI of −600 mV_Eocp_ vs. SCE both for 30 min of deposition time.

**Figure 6:**
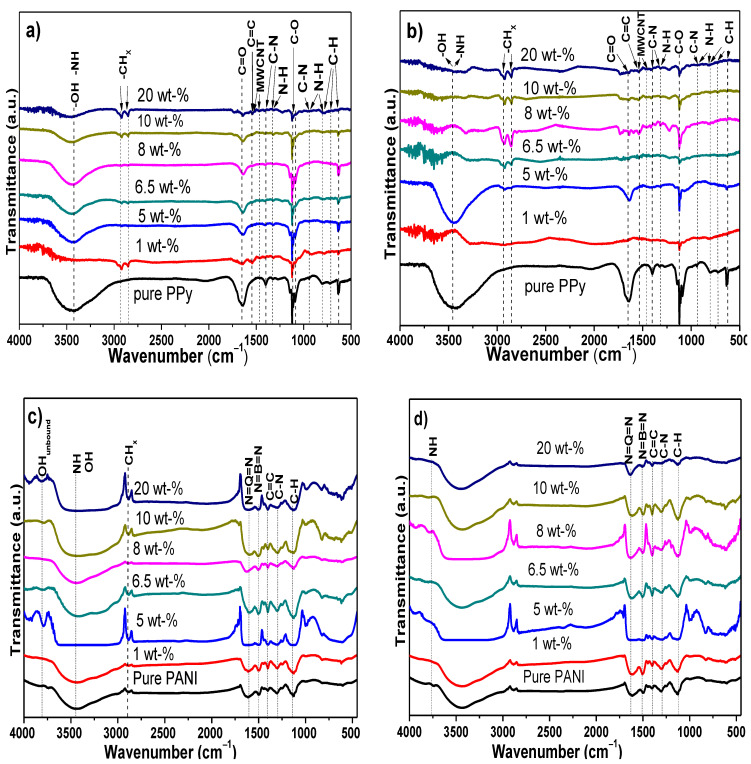
FT-IR spectra acquired from (**a**) comparison of PPy/MWCNTs nanocomposites with pure PPy; (**b**) PPy/commercial MWCNTs nanocomposites, (**c**) comparison PANI/MWCNT nanocomposites with pure PANI; (**d**) PANI/commercial MWCNTs nanocomposites.

**Figure 7:**
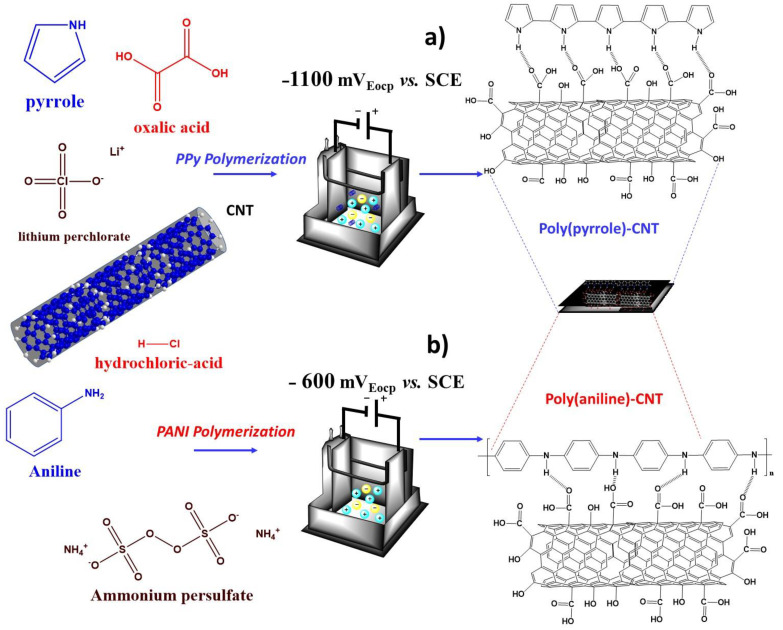
Physical interactions proposed for electropolymerized composites between (**a**) PPy, (**b**) PANI, and MWCNTs.

The chemical interaction of the films was initially studied by FT-IR measurements (Figure 6a–d). Pure PPy displays the characteristic bands at 3436 cm^−1^ correlated to the -NH stretching vibration mode of PPy [97,98]; C–N–C ring in-plane deformation and C=C stretching of PPy ring are distinguished at 1641 cm^−1^. C–N stretching and N–H ring in-plane bending are located between 1325 and1396 cm^−1^. Typical C-C stretching in-ring and C–N stretching are found at 1266 cm^−1^. Additionally, the C=C–N of ring in-plane deformation, C–N stretching, and N–H ring in-plane bending are at 1125 and 944 cm^−1^, respectively. A combination of C=C–N ring in-plane deformation, C–C=C inter-ring in-plane bending; C–C in-ring stretch, and C–C–N inter-ring in-plane bending, can be located at 1085 cm^−1^. Bands in the wavenumber range from 800 to 805 cm^−1^ correspond to N-H ring out-of-plane and C–H ring out-of-plane bending. The bands at 730 cm^−1^ show the C-H ring out-of-plane bending, whereas –CH flexion of cyclic plane can be observed at 633 cm^−1^ [97,99]. Some differences that can be observed in the electrodeposited PPy/MWCNTs composites are the typical O–H bands at 3300 and 3700 cm^−1^ that are overlapped with the –NH band. Additionally, the stretching vibrations of CH_x_ groups appear in the wavenumber range of 2900–2800 cm^−1^. Finally, the vibrational modes associated uniquely to MWCNTs appear at 1445 and 1575 cm^−1^ [68,69]. It is evident that a physical interaction occurs between MWCNTs and PPy, since they do not interfere in the individual bands. Similar interaction was observed in each composition when commercial MWCNTs were used for the synthesis of composites. Then, the most probable interactions between MWCNTs and PPy are physical. These possibly consisted of hydrogen bonding between the OH in MWCNTs with the NH groups in PPy, such as the R-NH…O=C–R and R–NH…H–O–R. Additionally, it is possible a contribution between the double bonds in both phases (known as π-π interactions); these interactions are proposed in Figure 7.

On the other hand, the FT-IR spectrum of pure PANI displays the following bands: NH stretching vibration of secondary amine overlapped with the free and hydrogen bonded NH at 3440 cm^−1^, C–H bending of benzenoid ring at 2890 cm^−1^, C=C vibrations of quinoid units, quinoid ring-stretching at 1500 cm^−1^, bending of CH in CH_2_ at 1452 cm^−1^, N–H bending mode at 1400 cm^−1^, CN stretching in benzene and quinoid rings at 1305 cm^−1^, CH bending at 1250 cm^−1^ and 1000 cm^−1^, C–H in-the-plane at 1130 cm^−1^, electronic transition of electrons in PANI structure at 1100 cm^−1^, C–H in-plane bending at 1000 cm^−1^, and the CH deformation of 1,4-disubstituted ring/Q ring at 826 cm^−1^ [89,100,101,102,103].

The spectra of electrodeposited PANI/MWCNTs showed an increase in the intensity in the bands of the functional groups that they have in common; one example is the overlap of OH in carboxylic groups adhered to the surface of CNTs as well as the NH stretching in the CN bands. The presence of some low intensity bands when MWCNTs are added in 5 wt% and 20 wt% into the polymer matrix matched with the unbounding hydroxyl groups. Based on this evidence, it is clear that the MWCNTs also could be interacting electrostatically with the PANI through the same interactions as PPy, i.e., NH…O=C–R and R–NH…H–O–R and π-π interactions (see Figure 7). The FT-IR spectra of conductive polymers (PANI or PPy) with commercial MWCNTs show bands and interactions quite similar to those observed with the as-synthesized MWCNTs.

**Figure 8:**
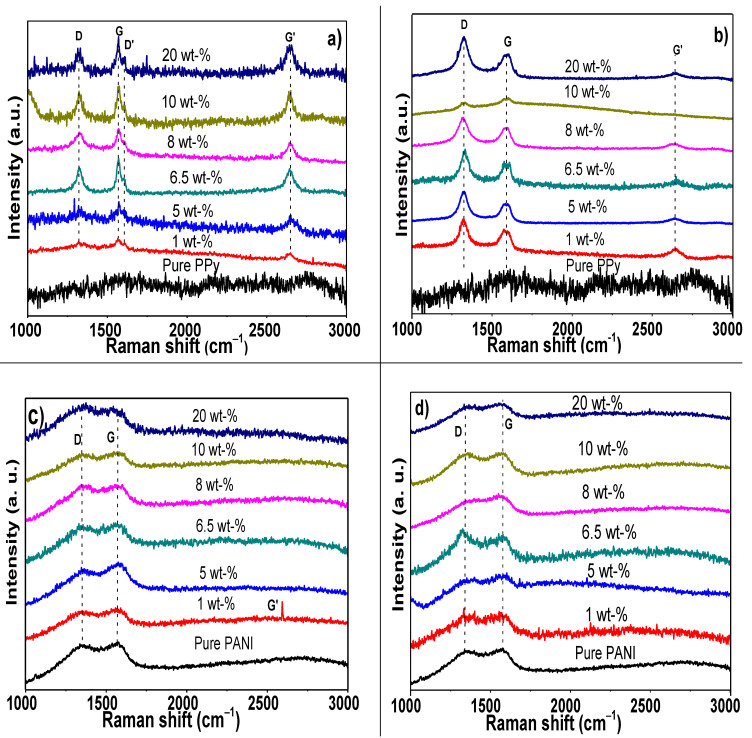
Raman of electropolymerized composites of (**a**) PPy/synthesized CNTs, (**b**) PPy/commercial MWCNTs nanocomposites, (**c**) PANI/synthesized MWCNTs, and (**d**) PANI/commercial MWCNTs.

In order to complement the FT-IR studies and evaluate the effect of electropolymerizing the PPy and PANI in presence of different wt% of MWCNTs, as well as to analyze the disorder and defects in crystal structure, Raman spectra were evaluated and presented in Figure 8. Pure PPy spectrum displayed a broad band c.a. 1600 cm^−1^ that corresponds to the C=C bonds and the inter-ring C–C mode of polaron structure, which evolves from the overlapping of neutral, oxidized (polaronic), and fully oxidized (bipolaronic) species located at 1560, 1580, and 1610 cm^−1^ [104].

The Raman spectra of the electropolymerized PPy composites with different compositions of MWCNTs synthesized at 1123 K and purified with HCl + H_2_O_2_ are shown in Figure 8a. A comparison of PPy/MWCNTs composites under similar synthesis conditions is presented in Figure 8b.

Raman spectra show that the bands more prominent at 1326 cm^−1^ (G), 1574 cm^−1^ (D), 1608 cm^−1^ (D’), and 2649 c^−1^ (G’) seem to correspond to MWCNTs, although some overlapping in the D and G bands of PPy cannot be discarded. An increase in the intensity of Raman bands with the amount of MWCNTs is observed. The I_D_/I_G_ ratio for polymer composites was computed from intensities, and the results are shown in Table 2. The I_D_/I_G_ ratio is very useful to analyze the extent of defects, and it has also been reported to be sensitive to molecular interaction at the interface [105].

The PPy/MWCNTs composites display a slight increase in this ratio when the samples are electropolymerized with low amounts of carbonaceous structures, 1 wt% (0.953) and 5 wt% (0.903), whereas with higher addition from 6.5 (0.745) to 20 wt%, this ratio decreases until 0.692. It is well known that when I_D_/I_G_ is closed to zero, it indicates a better structural order; specifically, in this case, it suggests a better molecular interaction between PPy-MWCNTs. I_D_/I_G_ ratio is quite different in the samples with the commercial CNTs, where a great quantity of disorder is obtained, since this ratio varies from 1.44 to 1.67; however, the composites still display a low quantity of defects (I_G’_/I_G_), Table 2. The increase in the I_D_/I_G_ can be related to a diameter of commercial of CNTs and poor interaction with PPy during electropolymerization process. Then, the behavior of I_D_/I_G_ ratio suggests that a better interaction occurs with the as-prepared samples with an adequate electrostatic interaction with amounts above 6.5 wt%.

On the other hand, PANI spectra showed two main contributions, the first at 1350 cm^−1^ corresponding to the stretching vibrational modes of the charged nitrogen segments, i.e., the C–N^+.^ stretching vibrations of delocalized polaronic structures, and the second at 1570 cm^−1^, corresponding to the C–C- stretching of B and Q and C=N [106], Figure 8c,d. In the case of the electropolymerized PANI/MWCNTs composites obtained by CVD, it was observed that the Raman bands of the MWCNTs are overlapped with those of the PANI (Figure 8c). The I_D_/I_G_ ratio in PÄNI composites is low, with a small percentage of MWCNTs, and it increased from 6.5 wt%. The general tendency in the case of composites with commercial CNTs is a reduction in the I_D_/I_G_ ratio (Figure 8d and Table 2). Most of the samples with exception of 8 wt% of synthesized and 1 and 6.5 wt% of commercial MWCNTs and incorporated into PANI had lower I_D_/I_G_ ratio than that reported by Shao et al. [107], (I_D_/I_G_= 1.08) and Thakur et al. (I_D_/I_G_ = 1.14) [108], for PANI grafted on the surface of MWCNTs and PANI/CNT, respectively. These results highlight that the optimization of CVD parameters helps to reach a better structural order and it can modify the interaction-type between PPy and functionalized MWCNTs [109].

### 3.3. UV-Visible Optical Absorption Studies of PPy/MWCNTs, PANI/MWCNTs Nanocomposites

**Figure 9:**
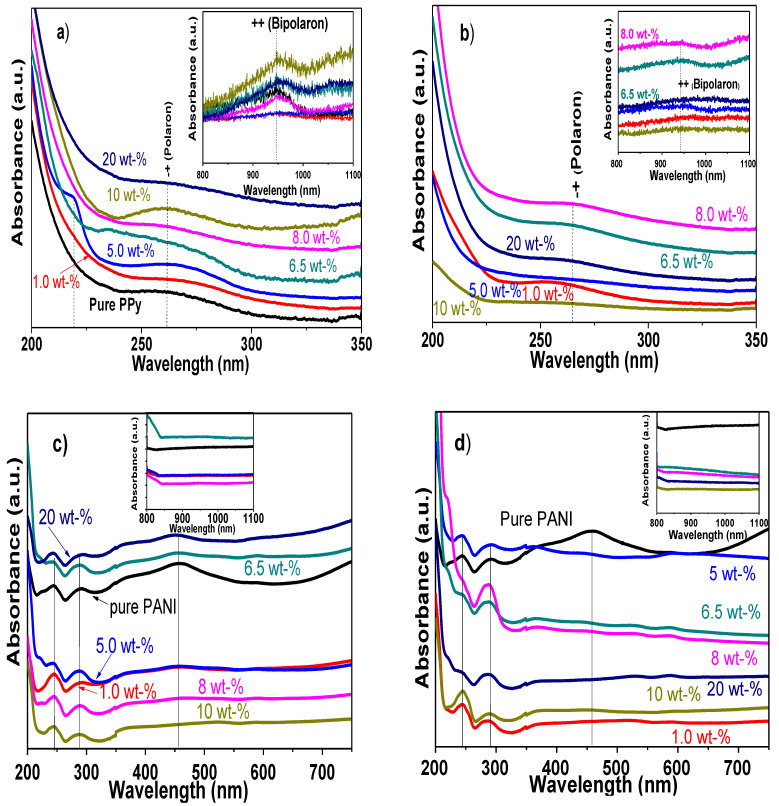
UV-Vis spectra from: (**a**) PPy/synthesized MWCNTs, (**b**) PPy/commercial MWCNTs nanocomposites, (**c**) PANI/synthesized CNTs, and (**d**) PANI /commercial MWCNTs nanocomposites. Pure PPy and PANI films are included as comparison.

The ultraviolet-visible spectra of electropolymerized samples were analyzed to evaluate the electron states of the pure PPy and PANI polymers, as well as to corroborate the interfacial interaction in the composites with MWCNTs. Figure 9a,b shows the UV-vis spectra of PPy/MWCNTs composites using synthesized and commercial MWCNTs, respectively.

Two main peaks were observed in the wavelength intervals of 200–350 nm and 800–1100 nm. The first peak ca. 260–270 nm is recognized to the polaron state coming from PPy and MWCNTs [110], whereas the second peak at approximately 938–948 nm corresponds to the bipolaron state of PPy (insets in Figure 9a,b) [111]. The polaron arises from the conjugated electron loss of pyrrole; then, polarons are linked together to form bipolaron during PPy polymerization [112]. These bands for pure PPy show a wide weak absorption band between 247–300 nm and a weak peak at ~947 nm (Figure 9a). In the case of nanocomposites, the peaks present small displacements towards a low wavelength as well as an increase in the intensity as function of the MWCNTs content in the CP matrix. Additionally, only the sample with 5 wt% synthesized MWCNTs shows a characteristic peak at 219 nm of MWCNTs correlated to π-π* transition of aromatic C–C bonds of the carbon network [20]. In comparison with the composites produced with commercial CNTs, it is observed that a reduction in the intensity of both polarons supporting that interaction and molecular order were better for CVD synthesized CNTs. In fact, some transitions, such as that observed at 219 nm (5 wt%), are missing in Figure 9b. It has been stated that modifications in intensity and position of PPy adsorption bands indicate variations in electronic structure and relocation of polaron levels due to the interaction with MWCNTs [113]. The small displacements observed in the absorption spectra also indicate the physical interaction of PPy and MWCNTs, where structure of conductive PPy is modified after the fabrication of nanocomposites. The results are in good agreement with previous reports of PPy nanocomposites, highlighting that with this conductive polymer, the interaction with other nanostructures occurs quite similarly [17,114].

On the other hand, Figure 9c,d displays the UV-visible spectra of PANI//MWCNTs composites using CVD and commercial CNTs. It is also included the UV-vis spectra of pure PANI. The spectra of pure PANI display three absorption maximums located at 243, 291, and 458 nm. The first two correspond to the π-π* transitions within the benzenoid ring (B), and the third is due to the polaron-π* transitions within doped quinoid (Q) structure in doped PANI [115,116]. The spectra of PANI/MWCNTs composites, using the synthesized carbonaceous structures, display absorbance spectra similar to an overlapping of the contribution of CNTs and PANI polymers between 243–291 nm.

The most important difference to highlight is that the polaron-π* transition tends to disappear after nanocomposite electropolymerizing, which is related to the interaction through the MWCNTs carbonyl groups. Similar absorbance spectra were obtained at any composition, when commercial MWCNTs were used to obtain composites; however, in all the spectra, the π-polaron transition is missing. Thus, by comparing the UV-vis spectra PANI nanocomposites using both MWCNTs types, it was observed that samples with high absorbance are those fabricated with an amount of 6.5 and 20 wt%; whereas for commercial CNTs, the samples that display the maximum absorbance values were with 5.0 and 6.5 wt%. In addition, the signal that corresponds to polaron-π* transitions within doped quinoid (Q) structure remains with nanocomposites prepared with CVD CNTs, but it disappears with commercial ones, highlighting a better interaction of the composites with the method used in this study. It is also important to recall that under these conditions, the conductive phase of PANI remains unaltered in the UV region after electropolymerization process [117]. Recent reports of PANI nanocomposites have demonstrated that the shift of optical absorption band maximum depends on the composition of polymerization bulk solution and polymerization process, which in turn modified the appearance of π-π* and polaron π-transitions [114], but in our case, such displacements were missing.

Finally, during the acid functionalization of MWCNTs, different -OH and -COOH groups are attached at the open ends and defect sites (Figure 7), and such interaction could be either covalent bonds or by NH…O=C–R, R–NH…H–O–R and/or π-π interactions. The UV-vis spectra confirm that a weak interfacial interaction between these groups occurs in both kinds of nanocomposites.

### 3.4. Electrical Conductivity Measurements

A four-point probe method was used to determine the electrical resistance and conductivity (δ) of pure PPy, PANI, and composites in pellet shape, at room temperature. Pure PPy showed an electrical conductivity of 1.16 × 10^−2^ ± 8.49 × 10^−2^ S cm^−1^, whereas the base-PPy nanocomposites tended to increase the electrical measurements with the amount of CNTs (Table 3). However, there is not a direct correlation between the electrical conductivity and the quantity of CNTs in the matrix. The composites that showed the highest values were those with a content of 6.5 wt% (12.5 ± 1.90 S cm^−1^), followed by 10 wt% (5.5 × 10^−1^ ± 1.15 × 10^−2^ S cm^−1^) and 20 wt% (5.0 × 10^−1^ ± 0.0 S cm^−1^). The other samples showed an uncertain contribution of the CNTs to the improvement of electrical conductivity.

On the other hand, pure PANI showed values 1.10 × 10^−2^ ± 8.80 × 10^−4^ S cm^−1^ whilst, in the case of PANI nanocomposites, the electrical conductivity presented a gradual enhance with the carbonaceous structures quantity. In this case, high conductivities were observed for samples with 20 wt% of CNTs (2.5 ± 0.0 S cm^−1^). The increase in the electrical conductivity of both polymers nanocomposites is commonly attributable to the π-π stacking between PPy or PANI and CNTs providing easier conductive channels. The differences between both polymers are due to the number of functional groups that lead to electrons transfer in a more efficient way between carbon atoms [118], which in turn is more effective in PPy than PANI matrix. The percolation behavior of CNTs in a PPy matrix has been found above 8.4 wt% of CNTs, depending on the synthesis parameters [109,118], but in this case, the best condition was attained with 6.5 wt% for PPy and 20 wt% for PANI. These differences at room temperature can be related to the dispersion during the pellet fabrication and temperature operation.

**Figure 10:**
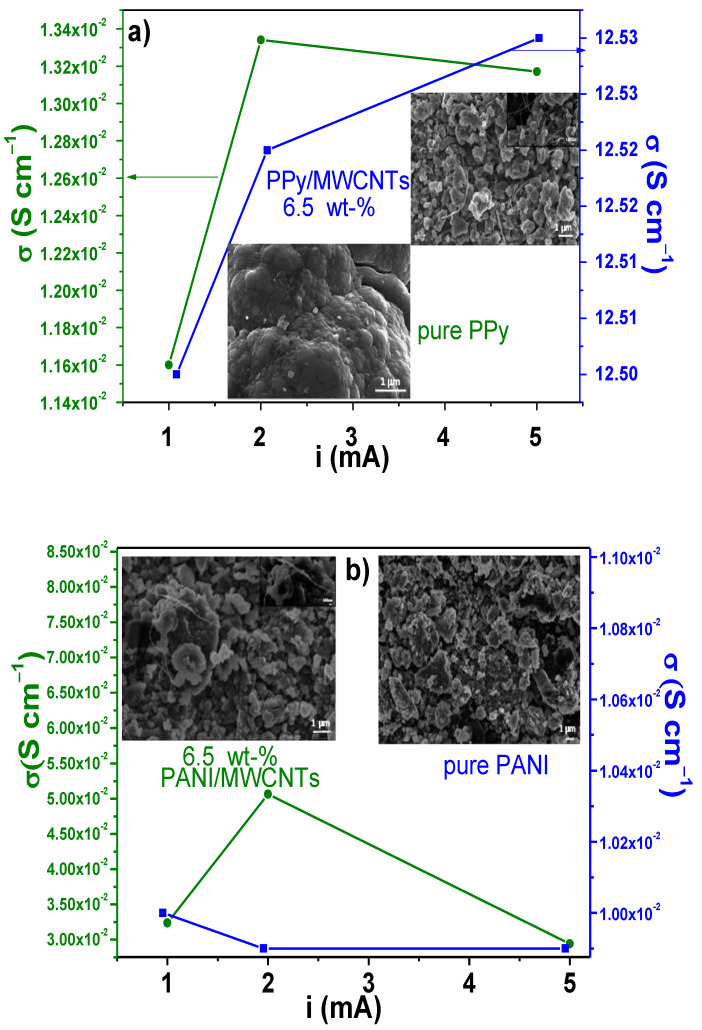
Variation of electrical conductivity performance of (**a**) PPy/MWCNT and (**b**) PANI/MWCNT composites at different applied currents.

To observe the changes in the electrical behavior with the applied current, nanocomposites with an amount of 6.5 wt% were evaluated by the four-point probe method using 1, 2, and 5 mA, Figure 10a,b. The nanocomposites were compared to pure matrixes (PPy or PANI). Figure 10a shows that the pure PPy conductivity increases from 1.16×10^−4^ ± 8.49 × 10^−4^ S cm^−1^ to 1.33 × 10^−2^ ± 2.1 × 10^−4^ S cm^−1^ with 1 and 2 mA, respectively. Thereafter, the electrical conductivity reduces using a current of 5 mA (1.31 × 10^−2^ ± 1.23 × 10^−3^ S cm^−1^), which can be due to the structural changes during the applied current.

The analyzed PPy/MWCNTs nanocomposites with 6.5 wt% present a fairly constant conductivity between 12.5 ± 1.90 and 12.53 ± 1.23 S cm^−1^. The result indicates that an adequate synergy with electrical stability has been reached between the MWCNTs and PPy [119,120]. Electrical conductivity of PANI and its nanocomposites (6.5 wt%) are shown in Figure 10b. In this figure, the pure PANI shows a similar trend as PPy with a reduction in the conductivity from 1.10 × 10^−2^ ± 8.80 × 10^−4^ to 9.9 × 10^−3^ ± 3.18 × 10^−4^ S cm^−1^; the lowest value is observed at 5 mA. It is possible that the emeraldine phase of PANI changes its oxidizing degree, and then it reduces the electrical conductivity. On the other hand, PANI composites again showed a slight increase with the electrical conductivity from 3.24 × 10^−2^ ± 6.48 × 10^−4^ to 5.06 × 10^−2^ ± 2.15 × 10^−4^ S cm^−1^ by varying the applied current from 1 to 2, but again they were reduced up to 2.94×10^−2^ ± 7.71 × 10^−4^ S cm^−1^ with 5 mA. These results clearly indicate that after this applied current, the composite loses its electrical stability.

In comparison with other composites such as PPy/perovskite manganite (5.0 × 10^−3^ S cm^−1^) [120], it can be seen that the electrical conductivity values of either PPy or PANI/MWCNTs composites were high enough. On the contrary, *σ* values of the PPy/MWCNTs and PANI/MWCNTs are low compared to previous reports in the literature, which display values in the range of 40–60 S cm^−1^ for PPy composites [121,122] and 32–223 S cm^−1^ for PANI composites depending of the CNT amount [27], but it is important to consider other factors such as MWCNTs quantity, pellet thickness, and compaction pressure. In fact, in the supplementary material (Figure S2 in the Supplementary Material), it can be observed that under similar conditions of synthesis, the electrical conductivity of PANI composites, using the synthesized nanotubes of this work, was high compared to the commercial source of CNTs. The DC conductivities of pure polymers (PPy or PANI) and their composites, at room temperature, are related to hopping of charge carriers between polymer chains and MWCNTs; PPy and PANI, being conjugated polymers, exhibit the hopping process in DC conductivity [120].

It has been reported that the conductivity in CPs varies with their morphologies and synthesis methods. The morphology observed in this work is presented as inset figures (Figure 10a,b). Differences in morphologies can be seen in a way that influence the measured electrical conductivities. In this context, for example, PPy nanotubes display 32 S cm^−1^ [123], whereas PPy globular powders show values from 1.55 to 2.11 S cm^−1^ [124]; PPy, produced by pyrrole oxidation with iron (III) chloride, exhibits values from 1.5 to 4.4 S cm^−1^ [125] and from 0.007 to 0.012 S cm^−1^ when PPy is prepared with a surfactant [126]. Similarly, the electrical conductivity of PANI doped with HCl in presence of ((NH_4_)_2_S_2_O_8_), as oxidant, has been reported to be 0.143 S cm^−1^ [127].

### 3.5. Hall Measurements

Hall effect measurements were also realized in these samples to determine the carrier density (*n*) and mobility (*μ*) at 300 K, using a Hall field of 0.550 T orthogonal to the current density. The relevance of Hall effect measurements is described in the literature [128]. The results for pure PPy, PANI, and selected sample (composite with 6.5 wt% MWCNTs), were as follows: for pure polymers PPy, *n* = 6.4 × 10^16^ ± 5.8×10^14^ cm^-3^, and *μ* = 1.9 ± 0.1 cm^2^ (V s)^−1^; in the case of pure PANI, *n* = 2.9 × 10^17^ ± 2.4 × 10^14^ cm^−3^ and *μ* = 17.9 ± 0.3 cm^2^ (V s)^−1^. Polymer nanocomposites with PPy displayed values of *n* = −1.6 × 10^19^ ± 1.6 × 10^17^ cm^−3^ and *μ* = 1.6 × 10^−2^ ± 1.3 × 10^−4^ cm^−3^ cm^2^ (V s)^−1^, whereas *n* = 4.9 × 10^18^ ± 6.1 × 10^16^ cm^−3^ and *μ* = 1.6 × 10^−2^ 2.1 × 10^−4^ cm^2^ (V s)^−1^ were obtained for PANI composites. Conventionally, the positive carriers are “holes”, and the negative carriers are electron in a semiconductor material (*n* values). Then, it can be observed that pure PPy presents an acceptor character, which is modified to donator character after adding the MWCNTs. In the case of pure PANI and PANI composites, both samples show an acceptor character [129]. The carrier densities of PPy nanocomposites increase two orders of magnitude and PANI increases in one order, highlighting that CNTs made an important difference in the conductive properties. Conversely, Hall mobility is reduced in both nanocomposites with values close to zero, indicating the possibility of quantum confinement of the charge carrier by the addition of CNTs; thus, it becomes more conductive. This final observation confirms that depending on the interaction between MWCNTs and polymer matrix, the carrier density and mobility can be modified. The choice of an adequate composition of CNTs in a conductive polymeric matrix promotes these properties, but they depend on the stability and morphology of the composites.

### 3.6. Structural Changes and Stability of Nanocomposites

**Figure 11:**
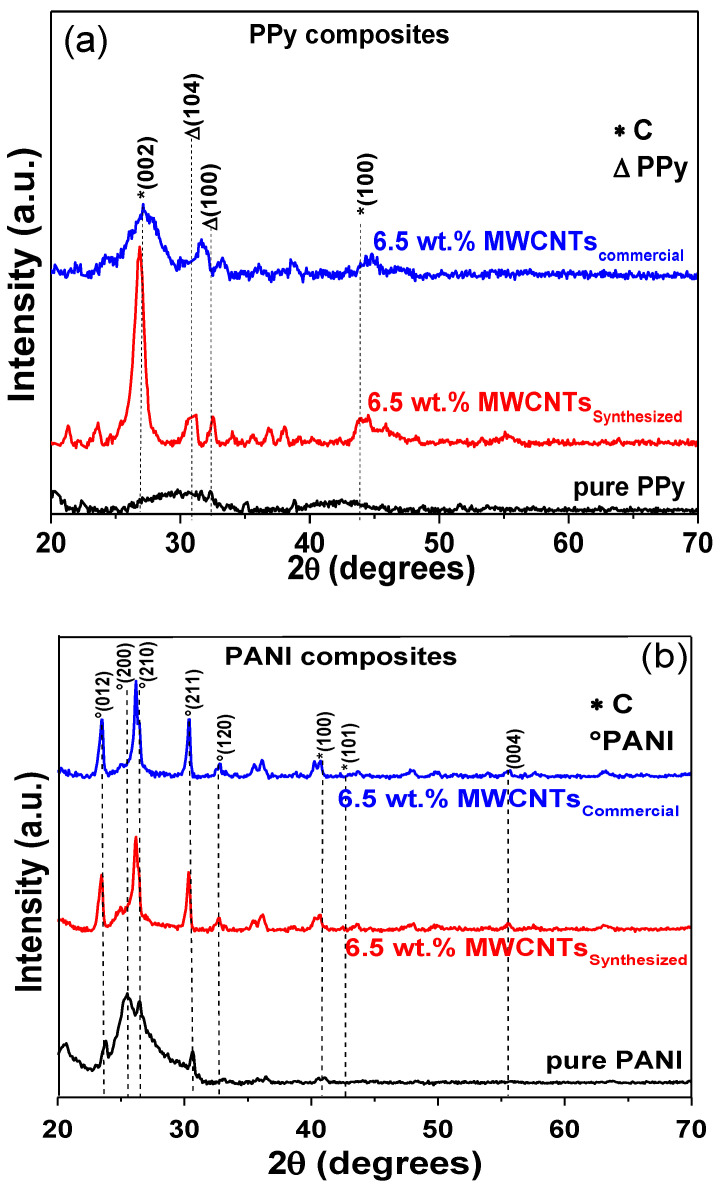
XRD patterns of selected (**a**) PPy/MWCNTs and (**b**) PANI/MWCNTs nanocomposites and their corresponding comparison with pure matrix and commercial MWCNTs.

To determine structural modifications of both polymer composites with the addition of CNTs (6.5 wt%) and after electrical characterization, XRD measurements were realized using synthesized and commercial MWCNTs. The comparison with pure polymer matrixes is also presented in the spectra. XRD patterns of pure PPy display broad signals, which are typical of amorphous phases [20,130], Figure 11a. The main diffraction peaks for commercial and synthesized MWCNTs are observed ca. 26°, 43°, and 45°, corresponding to the (002), (100), and (101) planes. The composites show crystalline structures induced by the addition of CNTs. An important observation in the synthesized MWCNTs is that the diffraction peaks become narrow, intense, and more defined in comparison to composites using commercial CNTs. As a result, the calculated crystallinity of the PPy/WMCNTs_commercial_ was of 58%, whereas PPy/MWCNTs_synthesized_ was about 78%. This increase in crystallinity in comparison to pure could be due to defects present in the composite, which is better using synthesized carbon nanotubes [131].

Pure PANI patterns display the characteristic signal of the (200) basal plane of the orthorhombic structure at 25.5°; the amplitude is indicative of the amorphous nature in this polymer (Figure 11b). Other signals are identified at 23.8°, 26.4°, 30.5°, and 32.6° corresponding to (012), (210), (211), and (120) (PDF# 53–1890). The XRD pattern of MWCNTs synthesized displays the most intense signal of graphite at 26.1°. As is observed in the patterns, the PANI composites with either synthesized or commercial MWCNTs display the contributions of both phases. The main effect after adding CNTs is again the increase in the crystallinity, with values of 47% and 36% for both composites prepared with commercial MWCNTs and CVD process. The structural order of the conductive polymers improves in the presence of MWCNTs, especially using CVD CNT. The physical interactions, the alignment, and defects are the responsibility of the improvement in the crystallinity degree. The results indicate that the nanocomposites are stable in the structure and crystallinity after the electric polarization.

After the conjugated electron loss of pyrrole, polarons are linked together to form bipolaron during PPy polymerization [112]. 12. As is reported, pure PPy displays three weight loss stages [132,133], (Figures S3 and S4 in the Supplementary Material). The first stage is attributed to the water removal (~13%, below 363 K); the second step is related to the volatile compounds in PPy (~18%, 363–514 K); and the final stage is characterized by a marked drop starting at 514 K and ending at 897 K (~69%), which indicates that after this temperature, PPy is totally decomposed [134,135]. In the case of pure PANI, the degradation process occurs in four stages: the first weight loss is correlated with the elimination of absorbed moisture (below 373 K, ~13%), followed by the weight loss of water molecules that are attached as a secondary bonds in PANI (403–633 K, ~14%). The third stage is due to the carbonization of PANI backbone, and it occurs between 673–953 K (~32%), and the last stage between above 953 K is correlated with the carbon burning (~41%).

**Figure 12:**
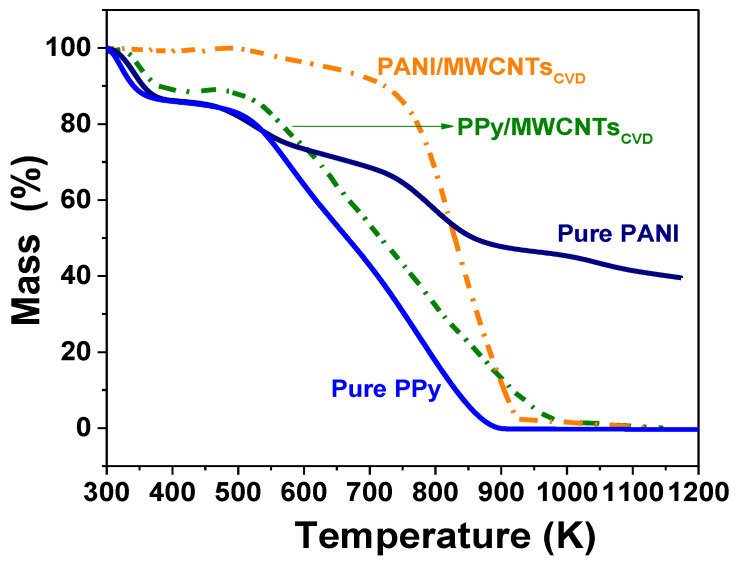
TGA thermograms of PPy/MWCNTs, PANI/MWCNTs composites using synthesized carbonaceous structures and their comparison with pure polymers.

PPy/MWCNTs composites (Figure 12) display a thermal behavior similar to pure PPy, but the decomposition requires a high temperature at each stage: <383 K (~10%), 383–523 K (~4%), and the third step from this temperature up to 996 K (~84%), confirming the improvement in the thermal properties due to addition of MWCNTs [105,133]. The balance is attributed to the carbon burning (~2%).

On the other hand, the TGA for PANI/MWCNTs (Figure 12) composites display an improvement in the thermal stability in comparison to the PPy/MWCNTs system. The first loss occurs from 298 K to 542 K (~2%); the second mass loss occurred in the temperature range of 542–741 K (~10%), and it is correlated with the elimination of moisture attached as secondary bonds. About 86% of weight loss occurs between 741 and 924 K due to total decomposition of PANI/MWCNTs composites [136]. Finally, about 3% is due to the residual carbon.

From the above results, it was found that the optimal conditions for synthesizing CVD CNTs modulate the stability, structural properties, and electrical features, which are essential for technological applications such as thermoelectric devices.

## 4. Conclusions

In this work PPy/MWCNTs and PANI/MWCNTs composites were synthesized by electropolymerization process to determine changes in their stability and electrical properties. The conditions during the synthesis of MWCNTs by CVD process such as the decomposition time, argon fluxes, amounts of precursor, temperature (at 1023 K and 1123 K), and the purification process (HNO_3_ + H_2_SO_4_ or HCl + H_2_O_2_) were also optimized and compared with commercial CNTs.

The results from structural and optical properties indicate that the main parameters influencing the purity of bamboo-like morphology CNTs are the processing temperature at 1123 K and the treatment with HCl + H_2_O_2_ that impacts directly on the elimination of Fe_3_C, as well as the increase of crystallinity degree and reduction of the absorption in UV-vis range. Under a similar purification process, the stability of the synthesized CNTs displayed an improvement in the thermal stability and structural order (I_D_/I_G_ ratio), but with high defects compared to commercial CNTs. PPy composites show similar electrostatic interactions, which are probably through hydrogen bonding, regardless of the MWCNTs amounts, but a better structural order was obtained from 6.5 wt%. In contrast, PANI/MWCNTs composites showed high structural order with low amounts of CNTs and increased after 6.5 wt%. The defects quantity (I_G’_/I_G_) of these samples is still higher than the observed in composites using commercial carbon nanotubes. The absorption bands of the PPy composites present the same transitions after polymerization process, whereas PANI/MWCNTs composites show that the polaron-π* transition is reduced after electropolymerizing. However, the conductive bands (polaron-π*) in the composites with commercial MWCNTs disappeared. Under these experimental conditions, an adequate amount to add them into the CPs matrixes was 6.5 wt%. The crystallinity of composites with optimized properties shows that the crystallinity degree and thermal stability related to the alignment of PANI and PPy chains on the MWCNTs are enhanced with the CNTs. Finally, in adequate amounts and synthesis parameters, the synthesized MWCNTs improve the carrier density and mobility of both CPs up to two orders in magnitude, which positively affect the electrical conductivity. Comparing both CPs, it can be concluded that the electrostatic interaction, stability, and conductivity are better in PPy/MWCNTs compared to PANI/MWCNTs composites.

## Figures and Tables

**Table 1:** Crystallite size and crystallinity percent for MWCNT synthesized at 1023 and 1123 K without and with purification. Samples at 1123 K were purified with two acid solutions (HCl + H_2_O_2_ and HNO_3_ + H_2_SO_4_) to determinate the effect of the crystallite size, crystallinity percent, and purity.

**CVD at 1023 K, Purified with HCl + H_2_O_2_**					
**Sample**	**2*θ*** **(deg)**	**t** **(nm), BP**	**t** **(nm),** **AP**	**d** **(nm),** **AP**	**Crystallinity** **(%)**
Commercial CNT	25.73	3.00	2.92	0.3459	85.30
W_0.1_F_50_t_30_	25.95	9.01	8.46	0.3431	88.67
W_0.1_F_50_t_60_	26.00	8.90	7.69	0.3424	91.36
W_0.1_F_100_t_30_	25.93	10.88	8.76	0.3433	97.78
W_0.1_F_100_t_60_	25.85	12.15	8.73	0.3443	97.03
W_0.2_F_50_t_30_	25.93	7.90	7.02	0.3433	97.54
W_0.2_F_50_t_60_	25.77	10.77	9.52	0.3454	96.96
W_0.2_F_100_t_30_	25.94	11.28	9.81	0.3432	91.54
W_0.2_F_100_t_60_	25.92	7.87	9.18	0.3434	98.07
**CVD at 1123 K, Purified with HCl + H_2_O_2_**					
Commercial CNT	25.73	3.00	2.93	0.3459	85.30
W_0.1_F_50_t_30_	25.82	12.42	8.73	0.3448	96.37
W_0.1_F_50_t_60_	25.93	11.59	8.64	0.3433	96.56
W_0.1_F_100_t_30_	25.88	10.75	8.55	0.3441	96.59
W_0.1_F_100_t_60_	25.88	12.41	9.04	0.3440	96.44
W_0.2_F_50_t_30_	26.02	10.06	8.30	0.3422	89.52
W_0.2_F_50_t_60_	26.00	10.40	8.63	0.3424	94.56
W_0.2_F_100_t_30_	25.94	13.81	8.47	0.3433	85.07
W_0.2_F_100_t_60_	25.92	10.69	9.41	0.3434	95.11
**CVD at 1123 K, Purified with HNO_3_ + H_2_SO_4_**					
W_0.1_F_50_t_30_	25.96	12.42	8.75	0.3429	94.20
W_0.1_F_50_t_60_	25.94	11.59	9.11	0.3432	84.41
W_0.1_F_100_t_30_	26.22	10.75	8.51	0.3396	96.21
W_0.1_F_100_t_60_	25.98	12.41	10.41	0.3427	86.83
W_0.2_F_50_t_30_	25.95	10.06	8.43	0.3431	95.45
W_0.2_F_50_t_60_	25.88	10.40	8.34	0.3439	95.38
W_0.2_F_100_t_30_	25.89	13.81	9.74	0.3438	95.21
W_0.2_F_100_t_60_	25.89	10.69	8.96	0.3438	87.32

BP: before purification; AP: after purification; t: crystallite size; d: interplanar distance.

**Table 2:** I_D_/I_G_ quotients compute from Figure 8, for PPy and PANI composites.

Content of MWCNTs	PPy/Synthesized MWCNTs	PPy/Commercial MWCNTs	PANI/Synthesized MWCNTs	PANI/Commercial MWCNTs
1 wt%	0.953	1.444	0.805	1.132
5 wt%	0.903	1.731	0.750	0.761
6.5 wt%	0.745	1.183	0.869	1.096
8 wt%	0.733	1.492	1.038	0.785
10 wt%	0.790	1.043	0.913	0.938
20 wt%	0.692	1.671	0.977	0.923

**Table 3:** Resistivity and electric conductivity of pure polymeric matrix (PPy or PANI) and nanocomposites with different MWCNTs amounts using a DC current of 1 mA, at room temperature. For these measurements, it was considered the surface area of each pellet.

Sample	Ω*cm	S cm^−1^	Sample	Ω*cm	S cm^−1^
PPy	86.3 ± 0.6	1.16 × 10^−2^ ± 8.49 × 10^−4^	PANI	90.6 ± 7.2	1.10 × 10^−2^ ± 8.80 × 10^−4^
1 wt%	35.4 ± 0.4	2.82 × 10^−2^ ± 3.90 × 10^−4^	1 wt%	64.4 ± 14.1	1.55 × 10^−2^ ± 3.41 × 10^−3^
5 wt%	15.3 ± 0.0	6.5 × 10^−2^ ± 0.0	5 wt%	55.3 ± 18.8	1.80 × 10^−2^ ± 5.94 × 10^−3^
6.5 wt%	0.08 ± 0.01	12.5 ± 1.90	6.5 wt%	30.8 ± 0.6	3.24 × 10^−2^ ± 6.48 × 10^−4^
8 wt%	10.9 ± 0.0	9.1 × 10^−2^ ± 0.0	8 wt%	8.3 ± 0.2	1.2 × 10^−1^ ± 3.6 × 10^−3^
10 wt%	1.8 ± 0.3	5.5 × 10^−1^ ± 1.15 × 10^−2^	10 wt%	3.8 ± 1.0	2.6 × 10^−1^ ± 7.28 × 10^−2^
20 wt%	2.0 ± 0.0	5.0 × 10^−1^ ± 0.0	20 wt%	0.4 ± 0.0	2.5 ± 0.0

## Data Availability

No new data were created or analyzed in this study. Data sharing is not applicable to this article.

## Supplementary Materials

The following are available online at https://www.mdpi.com/2073-4360/13/3/351/s1, Figure S1. UV-vis spectrum of purified commercial MWCNTs; Figure S2. Comparison of electrical conductivity of PANI composites using the synthesized and commercial nanotubes with an amount of 6.5 wt%; Figure S3. Derivative thermogravimetric analysis of PPy, MW carbon nanotubes synthesized and commercial; Figure S4. TGA thermograms of pure polymers.

## References

[1] Wang X.-X., Yu G.-F., Zhang J., Yu M., Ramakrishna S., Long Y.-Z. (2020). Conductive Polymer Ultrafine Fibers via Electrospinning: Preparation, Physical Properties and Applications. Prog. Mater. Sci..

[2] Dias O.A.T., Konar S., Leão A.L., Sain M. (2019). Flexible electrically conductive films based on nanofibrillated cellulose and polythiophene prepared via oxidative polymerization. Carbohydr. Polym..

[3] Matysiak W., Tański T., Smok W., Gołombek K., Schab-Balcerzak E. (2020). Effect of conductive polymers on the optical properties of electrospun polyacrylonitryle nanofibers filled by polypyrrole, polythiophene and polyaniline. Appl. Surf. Sci..

[4] Taghizadeh A., Taghizadeh M., Jouyandeh M., Yazdi M.K., Zarrintaj P., Saeb M.R., Lima E.C., Gupta V.K. (2020). Conductive polymers in water treatment: A review. J. Mol. Liq..

[5] Zhao P., Tang Q., Zhao X., Tong Y., Liu Y. (2018). Highly stable and flexible transparent conductive polymer electrode patterns for large-scale organic transistors. J. Colloid Interface Sci..

[6] Yang Z., Ma J., Bai B., Qiu A., Losic D., Shi D., Chen M. (2019). Free-standing PEDOT/polyaniline conductive polymer hydrogel for flexible solid-state supercapacitors. Electrochim. Acta.

[7] Li J., Ma Y. (2016). In-situ synthesis of transparent conductive PEDOT coating on PET foil by liquid phase depositional polymerization of EDOT. Synth. Met..

[8] Norouzian R.-S., Lakouraj M.M. (2020). Polyaniline-thiacalix[4]arene metallopolymer, self-doped, and externally doped conductive polymers. Prog. Org. Coat..

[9] Biswas M.R.U.D., Cho K.Y., Na J.D., Oh W.-C. (2019). Highly electro-conductive graphene-decorated PANI-BiVO4 polymer-semiconductor nanocomposite with outstanding photocatalytic performance. Mater. Sci. Eng. B.

[10] Zhang H., Sun X., Hubbe M.A., Pal L. (2019). Highly conductive carbon nanotubes and flexible cellulose nanofibers composite membranes with semi-interpenetrating networks structure. Carbohydr. Polym..

[11] Zamani P., Higgins D., Hassan F., Jiang G., Wu J., Abureden S., Chen Z. (2014). Electrospun Iron–Polyaniline–Polyacrylonitrile Derived Nanofibers as Non–Precious Oxygen Reduction Reaction Catalysts for PEM Fuel Cells. Electrochim. Acta.

[12] Anwane R.S., Kondawar S.B., Late D.J. (2018). Bessel’s polynomial fitting for electrospun polyacrylonitrile/polyaniline blend nanofibers based ammonia sensor. Mater. Lett..

[13] Bai H., Zhao L., Lu C., Li C., Shi G. (2009). Composite nanofibers of conducting polymers and hydrophobic insulating polymers: Preparation and sensing applications. Polymer.

[14] Kamble D.B., Sharma A.K., Yadav J.B., Patil V.B., Devan R.S., Jatratkar A.A., Yewale M.A., Ganbavle V.V., Pawar S.D. (2017). Facile chemical bath deposition method for interconnected nanofibrous polythiophene thin films and their use for highly efficient room temperature NO_2_ sensor application. Sens. Actuators B Chem..

[15] Jia X., Ge Y., Shao L., Wang C., Wallace G.G. (2019). Tunable Conducting Polymers: Toward Sustainable and Versatile Batteries. ACS Sustain. Chem. Eng..

[16] Ibanez J.G., Rincón M.E., Gutierrez-Granados S., Chahma M.H., Jaramillo-Quintero O.A., Frontana-Uribe B.A. (2018). Conducting Polymers in the Fields of Energy, Environmental Remediation, and Chemical–Chiral Sensors. Chem. Rev..

[17] German N., Ramanaviciene A., Ramanavicius A. (2020). Formation and Electrochemical Evaluation of Polyaniline and Polypyrrole Nanocomposites Based on Glucose Oxidase and Gold Nanostructures. Polymers.

[18] Barsan M.M., Enache T.A., Preda N., Stan G., Apostol N.G., Matei E., Kuncser A., Diculescu V.C. (2019). Direct Immobilization of Biomolecules through Magnetic Forces on Ni Electrodes via Ni Nanoparticles: Applications in Electrochemical Biosensors. ACS Appl. Mater. Interfaces.

[19] Farka Z., Juřík T., Kovář D., Trnková L., Skládal P. (2017). Nanoparticle-Based Immunochemical Biosensors and Assays: Recent Advances and Challenges. Chem. Rev..

[20] Li H., Liu S., Li P., Yuan D., Zhou X., Sun J., Lu X., He C. (2018). Interfacial control and carrier tuning of carbon nanotube/polyaniline composites for high thermoelectric performance. Carbon.

[21] Cho C., Wallace K.L., Tzeng P., Hsu J.-H., Yu C., Grunlan J.C. (2016). Outstanding Low Temperature Thermoelectric Power Factor from Completely Organic Thin Films Enabled by Multidimensional Conjugated Nanomaterials. Adv. Energy Mater..

[22] Toshima N. (2017). Recent progress of organic and hybrid thermoelectric materials. Synth. Met..

[23] Victorious A., Clifford A., Saha S., Zhitomirsky I., Soleymani L. (2019). Integrating TiO_2_ Nanoparticles within a Catecholic Polymeric Network Enhances the Photoelectrochemical Response of Biosensors. J. Phys. Chem. C.

[24] Barkauskas J., Mikoliunaite L., Paklonskaite I., Genys P., Petroniene J.J., Morkvenaite-Vilkonciene I., Ramanaviciene A., Samukaite-Bubniene U., Ramanavicius A. (2019). Single-walled carbon nanotube based coating modified with reduced graphene oxide for the design of amperometric biosensors. Mater. Sci. Eng. C Mater. Biol. Appl..

[25] Wongkaew N., Simsek M., Griesche C., Baeumner A.J. (2019). Functional Nanomaterials and Nanostructures Enhancing Electrochemical Biosensors and Lab-on-a-Chip Performances: Recent Progress, Applications, and Future Perspective. Chem. Rev..

[26] Zhang W., Ma J., Gao D., Zhou Y., Li C., Zha J., Zhang J. (2016). Preparation of amino-functionalized graphene oxide by Hoffman rearrangement and its performances on polyacrylate coating latex. Prog. Org. Coat..

[27] Xu R., Wei J., Guo F., Cui X., Zhang T., Zhu H., Wang K., Wu D. (2015). Highly conductive, twistable and bendable polypyrrole–carbon nanotube fiber for efficient supercapacitor electrodes. RSC Adv..

[28] Bauhofer W., Kovacs J.Z. (2009). A review and analysis of electrical percolation in carbon nanotube polymer composites. Compos. Sci. Technol..

[29] Matos M.A.S., Tagarielli V.L., Baiz-Villafranca P.M., Pinho S.T. (2018). Predictions of the electro-mechanical response of conductive CNT-polymer composites. J. Mech. Phys. Solids.

[30] Kazazi M. (2019). High-performance electrode based on electrochemical polymerization of polypyrrole film on electrophoretically deposited CNTs conductive framework for supercapacitors. Solid State Ion..

[31] Kazazi M., Vaezi M.R., Kazemzadeh A. (2014). Enhanced rate performance of polypyrrole-coated sulfur/MWCNT cathode material: A kinetic study by electrochemical impedance spectroscopy. Ionics.

[32] Zheng B., Li Y., Liu J. (2002). CVD synthesis and purification of single-walled carbon nanotubes on aerogel-supported catalyst. Appl. Phys. A Mater. Sci. Process..

[33] Ando Y., Zhao X., Sugai T., Kumar M. (2004). Growing carbon nanotubes. Mater. Today.

[34] Novoselova I., Oliinyk N.F., Volkov S., Konchits A., Yanchuk I., Yefanov V.S., Kolesnik S.P., Karpets M. (2008). Electrolytic synthesis of carbon nanotubes from carbon dioxide in molten salts and their characterization. Phys. E Low-Dimens. Syst. Nanostruct..

[35] Flamant G., Laplaze D. (2005). Solar synthesis of single-walled carbon nanotubes at medium scale. Carbon.

[36] Viculis L.M., Mack J.J., Kaner R.B. (2003). A chemical route to carbon nanoscrolls. Science.

[37] Chen Y., Conway M., Fitz gerald J., Williams J.S., Chadderton L.T. (2004). The nucleation and growth of carbon nanotubes in a mechano-thermal process. Carbon.

[38] Zhu Y., Lin T., Liu Q., Chen Y., Zhang G., Xiong H., Zhang H. (2006). The effect of nickel content of composite catalysts synthesized by hydrothermal method on the preparation of carbon nanotubes. Mater. Sci. Eng. B.

[39] Varshney D., Weiner B.R., Morell G. (2010). Growth and field emission study of a monolithic carbon nanotube/diamond composite. Carbon.

[40] Prasek J., Drbohlavova J., Chomoucka J., Hubalek J., Jasek O., Adam V., Kizek R. (2011). Methods for carbon nanotubes synthesis—Review. J. Mater. Chem..

[41] Domínguez-Crespo M.A., Torres-Huerta A., Onofre-Bustamante E., Alanis-Valdelamar A., Escudero M., Brachetti B. (2014). Corrosion studies of PPy/Ni organic–inorganic hybrid bilayer coatings on commercial carbon steel. J. Solid State Electrochem..

[42] Cabezas A.L., Zhang Z.-B., Zheng L.-R., Zhang S.-L. (2010). Morphological development of nanofibrillar composites of polyaniline and carbon nanotubes. Synth. Met..

[43] Duc Vu Quyen N., Quang Khieu D., Tuyen T.N., Xuan Tin D., Thi Hoang Diem B. (2019). Carbon Nanotubes: Synthesis via Chemical Vapour Deposition without Hydrogen, Surface Modification, and Application. J. Chem..

[44] Bhatia R., Prasad V. (2010). Synthesis of multiwall carbon nanotubes by chemical vapor deposition of ferrocene alone. Solid State Commun..

[45] Öncel Ç., Yürüm Y. (2006). Carbon Nanotube Synthesis via the Catalytic CVD Method: A Review on the Effect of Reaction Parameters. Fuller. Nanotub. Carbon Nanostruct..

[46] Yu J., Lucas J., Strezov V., Wall T. (2003). Coal and carbon nanotube production. Fuel.

[47] Maruyama S., Kojima R., Miyauchi Y., Chiashi S., Kohno M. (2002). Low-temperature synthesis of high-purity single-walled carbon nanotubes from alcohol. Chem. Phys. Lett..

[48] Zhao N., He C., Li J., Jiang Z., Li Y. (2006). Study on purification and tip-opening of CNTs fabricated by CVD. Mater. Res. Bull..

[49] Salaeh S., Thitithammawong A., Salae A. (2020). Highly enhanced electrical and mechanical properties of methyl methacrylate modified natural rubber filled with multiwalled carbon nanotubes. Polym. Test..

[50] Yu L., Shearer C., Shapter J. (2016). Recent Development of Carbon Nanotube Transparent Conductive Films. Chem. Rev..

[51] Min C., Shen X., Shi Z., Chen L., Xu Z. (2010). The Electrical Properties and Conducting Mechanisms of Carbon Nanotube/Polymer Nanocomposites: A Review. Polym. Plast. Technol. Eng..

[52] Dasari A., Yu Z.-Z., Mai Y.-W. (2009). Electrically conductive and super-tough polyamide-based nanocomposites. Polymer.

[53] Yang K., Han H., Pan X., Chen N., Gu M. (2008). The effect of chemical treatment on the crystallinity of multi-walled carbon nanotubes. J. Phys. Chem. Solids.

[54] Yuan J.-M., Chen X.-H., Chen X.-H., Fan Z.-F., Yang X.-G., Chen Z.-H. (2008). An easy method for purifying multi-walled carbon nanotubes by chlorine oxidation. Carbon.

[55] Han Z., Fina A. (2011). Thermal conductivity of carbon nanotubes and their polymer nanocomposites: A review. Prog. Polym. Sci..

[56] Azodpour J., Baniadam M. (2019). Microwave assisted purification of multi-walled carbon nanotubes by potassium permanganate; effect of acid to oxidant molar ratio and treatment time. Diam. Relat. Mater..

[57] Tan J.M., Karthivashan G., Arulselvan P., Fakurazi S., Hussein M.Z. (2014). Characterization and In Vitro Sustained Release of Silibinin from pH Responsive Carbon Nanotube-Based Drug Delivery System. J. Nanomater..

[58] Bankole M.T., Abdulkareem A.S., Mohammed I.A., Ochigbo S.S., Tijani J.O., Abubakre O.K., Roos W.D. (2019). Selected Heavy Metals Removal From Electroplating Wastewater by Purified and Polyhydroxylbutyrate Functionalized Carbon Nanotubes Adsorbents. Sci. Rep..

[59] Granados-Martínez F.G., Domratcheva-Lvova L., Flores-Ramírez N., García-González L., Zamora-Peredo L., Mondragón-Sánchez M.d.L. (2016). Composite Films from Polystyrene with Hydroxyl end Groups and Carbon Nanotubes. Mater. Res..

[60] Ahmed D.S., Haider A.J., Mohammad M.R. (2013). Comparesion of Functionalization of Multi-Walled Carbon Nanotubes Treated by Oil Olive and Nitric Acid and their Characterization. Energy Procedia.

[61] Hajighorbani M., Hekmati M. (2016). Pd nanoparticles deposited on Isoniazid grafted multi walled carbon nanotubes: Synthesis, characterization and application for Suzuki reaction in aqueous media. RSC Adv..

[62] Mohammadi M., Garmarudi A.B., Khanmohammadi M., Rouchi M.B. (2016). Infrared spectrometric evaluation of carbon nanotube sulfonation. Fuller. Nanotub. Carbon Nanostruct..

[63] Matranga C., Chen L., Smith M., Bittner E., Johnson J.K., Bockrath B. (2003). Trapped CO_2_ in Carbon Nanotube Bundles. J. Phys. Chem. B.

[64] Matranga C., Bockrath B. (2004). Permanent Trapping of CO_2_ in Single-Walled Carbon Nanotubes Synthesized by the HiPco Process. J. Phys. Chem. B.

[65] Osswald S., Havel M., Gogotsi Y. (2007). Monitoring oxidation of multiwalled carbon nanotubes by Raman spectroscopy. J. Raman Spectrosc..

[66] Ţucureanu V., Matei A., Avram A.M. (2016). FTIR Spectroscopy for Carbon Family Study. Crit. Rev. Anal. Chem..

[67] Garg P., Singh B.P., Kumar G., Gupta T., Pandey I., Seth R.K., Tandon R.P., Mathur R.B. (2011). Effect of dispersion conditions on the mechanical properties of multi-walled carbon nanotubes based epoxy resin composites. J. Polym. Res..

[68] Misra A., Tyagi P.K., Rai P., Misra D.S. (2007). FTIR spectroscopy of multiwalled carbon nanotubes: A simple approach to study the nitrogen doping. J. Nanosci. Nanotechnol..

[69] Kastner J., Pichler T., Kuzmany H., Curran S., Blau W., Weldon D.N., Delamesiere M., Draper S., Zandbergen H. (1994). Resonance Raman and infrared spectroscopy of carbon nanotubes. Chem. Phys. Lett..

[70] Njuguna J., Vanli O.A., Liang R. (2015). A Review of Spectral Methods for Dispersion Characterization of Carbon Nanotubes in Aqueous Suspensions. J. Spectrosc..

[71] Jiang L., Gao L., Sun J. (2003). Production of aqueous colloidal dispersions of carbon nanotubes. J. Colloid Interface Sci..

[72] Yu J., Grossiord N., Koning C.E., Loos J. (2007). Controlling the dispersion of multi-wall carbon nanotubes in aqueous surfactant solution. Carbon.

[73] Ryabenko A.G., Dorofeeva T.V., Zvereva G.I. (2004). UV–VIS–NIR spectroscopy study of sensitivity of single-wall carbon nanotubes to chemical processing and Van-der-Waals SWNT/SWNT interaction. Verification of the SWNT content measurements by absorption spectroscopy. Carbon.

[74] Mendoza-Cachú D., López-Miranda J.L., Mercado-Zúñiga C., Rosas G. (2018). Functionalization of MWCNTs with Ag-AuNPs by a green method and their catalytic properties. Diam. Relat. Mater..

[75] Meng J., Yang M., Song L., Kong H., Wang C.Y., Wang R., Wang C., Xie S.S., Xu H.Y. (2009). Concentration control of carbon nanotubes in aqueous solution and its influence on the growth behavior of fibroblasts. Colloids Surf. B Biointerfaces.

[76] Levitsky I.A., Euler W.B., Karachevtsev V.A. (2012). Photophysics of Carbon Nanotubes Interfaced with Organic and Inorganic Materials.

[77] He Z., Lan X., Chen F., Wang K., Deng H., Zhang Q., Fu Q. (2013). Effect of surface wettability on transparency in different water conditions. J. Coat. Technol. Res..

[78] Liu H., Zhai J., Jiang L. (2006). Wetting and anti-wetting on aligned carbon nanotube films. Soft Matter.

[79] Jeong S.H., Kim K.K., Jeong S.J., An K.H., Lee S.H., Lee Y.H. (2007). Optical absorption spectroscopy for determining carbon nanotube concentration in solution. Synth. Met..

[80] Lin M.F., Shung K.W.K. (1994). Plasmons and optical properties of carbon nanotubes. Phys. Rev. B.

[81] Rance G.A., Marsh D.H., Nicholas R.J., Khlobystov A.N. (2010). UV–vis absorption spectroscopy of carbon nanotubes: Relationship between the π-electron plasmon and nanotube diameter. Chem. Phys. Lett..

[82] Escobar B., Barbosa R., Miki Yoshida M., Verde Gomez Y. (2013). Carbon nanotubes as support of well dispersed platinum nanoparticles via colloidal synthesis. J. Power Sources.

[83] Zeferino González I., Valenzuela-Muñiz A.M., Gauvin R., Miki-Yoshida M., Verde-Gómez Y. (2020). Influence of the synthesis temperature and silicon concentration on the properties of Si doped MWCNT. Diam. Relat. Mater..

[84] Wang R., Wu H., Chen R., Chi Y. (2019). Strong Electrochemiluminescence Emission from Oxidized Multiwalled Carbon Nanotubes. Small.

[85] Dresselhaus M.S., Jorio A., Hofmann M., Dresselhaus G., Saito R. (2010). Perspectives on Carbon Nanotubes and Graphene Raman Spectroscopy. Nano Lett..

[86] Audiffred M., Elías A.L., Gutiérrez H.R., López-Urías F., Terrones H., Merino G., Terrones M. (2013). Nitrogen–Silicon Heterodoping of Carbon Nanotubes. J. Phys. Chem. C.

[87] Darne C., Desforges A., Berrada N., Fontana C., Guichard Y., Gaté L., Bégin D., Le Normand F., Valsaque F., Ghanbaja J. (2019). A non-damaging purification method: Decoupling the toxicity of multi-walled carbon nanotubes and their associated metal impurities. Environ. Sci. Nano.

[88] Zdrojek M., Gebicki W., Jastrzebski C., Melin T., Huczko A. (2004). Studies of Multiwall Carbon Nanotubes Using Raman Spectroscopy and Atomic Force Microscopy. Solid State Phenom..

[89] Mammadova S.A., Huseynov A.B., Israfilov A.O. (2018). Synthesis and Optical Properties of Iodinated Multi-walled Carbon Nanotubes. Opt. Spectrosc..

[90] Ezz A.A., Kamel M.M., Saad G.R. (2019). Synthesis and characterization of nanocarbon having different morphological structures by chemical vapor deposition over Fe-Ni-Co-Mo/MgO catalyst. J. Saudi Chem. Soc..

[91] Qi X., Zhong W., Deng Y., Au C., Du Y. (2010). Synthesis of helical carbon nanotubes, worm-like carbon nanotubes and nanocoils at 450 °C and their magnetic properties. Carbon.

[92] Cox B.J., Hill J.M. (2007). New Carbon Molecules in the Form of Elbow-Connected Nanotori. J. Phys. Chem. C.

[93] Zhao W., Shan C., Elias A.L., Rajukumar L.P., O’Brien D.J., Terrones M., Wei B., Suhr J., Lu X.L. (2015). Hyperelasticity of three-dimensional carbon nanotube sponge controlled by the stiffness of covalent junctions. Carbon.

[94] Vanyorek L., Sikora E., Balogh T., Román K., Marossy K., Pekker P., Szabó T.J., Viskolcz B., Fiser B. (2020). Nanotubes as polymer composite reinforcing additive materials—A comparative study. Arab. J. Chem..

[95] Pan L., Zhang M., Nakayama Y. (2002). Growth mechanism of carbon nanocoils. J. Appl. Phys..

[96] Baba M., Sano H., Zheng G.-B., Uchiyama Y. (2009). Effect of Mo in Co-Mo/MgO catalysts on the synthesis yield and structure of carbon nanotubes. J. Ceram. Soc. Jpn..

[97] Kostić R., Raković D., Stepanyan S.A., Davidova I.E., Gribov L.A. (1995). Vibrational spectroscopy of polypyrrole, theoretical study. J. Chem. Phys..

[98] Minisy I.M., Gavrilov N., Acharya U., Morávková Z., Unterweger C., Mičušík M., Filippov S.K., Kredatusová J., Pašti I.A., Breitenbach S. (2019). Tailoring of carbonized polypyrrole nanotubes core by different polypyrrole shells for oxygen reduction reaction selectivity modification. J. Colloid Interface Sci..

[99] Hien H.T., Van Tuan C., Anh Thu D.T., Ngan P.Q., Thai G.H., Doanh S.C., Giang H.T., Van N.D., Trung T. (2019). Influence of surface morphology and doping of PPy film simultaneously polymerized by vapour phase oxidation on gas sensing. Synth. Met..

[100] Neelgund G.M., Oki A. (2011). A facile method for synthesis of polyaniline nanospheres and effect of doping on their electrical conductivity. Polym. Int..

[101] Al-Hussaini A.S., Elias A.M., Abd El-Ghaffar M.A. (2017). New Poly(aniline-co-o-phenylenediamine)/Kaolinite Microcomposites for Water Decontamination. J. Polym. Environ..

[102] Karbownik I., Rac-Rumijowska O., Fiedot-Toboła M., Rybicki T., Teterycz H. (2019). The Preparation and Characterization of Polyacrylonitrile-Polyaniline (PAN/PANI) Fibers. Materials.

[103] de Araújo G.M., Codognoto L., Simões F.R. (2020). Self-assembled electrodes based on polyaniline grafted with reduced graphene oxide and polystyrene sulfonate. J. Solid State Electrochem..

[104] Trchová M., Stejskal J. (2018). Resonance Raman Spectroscopy of Conducting Polypyrrole Nanotubes: Disordered Surface versus Ordered Body. J. Phys. Chem. A.

[105] Zhang F., Xiao F., Dong Z.H., Shi W. (2013). Synthesis of polypyrrole wrapped graphene hydrogels composites as supercapacitor electrodes. Electrochim. Acta.

[106] Kumar V., Mahajan R., Bhatnagar D., Kaur I. (2016). Nanofibers synthesis of ND:PANI composite by liquid/liquid interfacial polymerization and study on the effect of NDs on growth mechanism of nanofibers. Eur. Polym. J..

[107] Shao D., Hu J., Chen C., Sheng G., Ren X., Wang X. (2010). Polyaniline Multiwalled Carbon Nanotube Magnetic Composite Prepared by Plasma-Induced Graft Technique and Its Application for Removal of Aniline and Phenol. J. Phys. Chem. C.

[108] Thakur A.K., Deshmukh A.B., Choudhary R.B., Karbhal I., Majumder M., Shelke M.V. (2017). Facile synthesis and electrochemical evaluation of PANI/CNT/MoS_2_ ternary composite as an electrode material for high performance supercapacitor. Mater. Sci. Eng. B.

[109] Saheeda P., Jayaleksmi S. (2020). Liquid/liquid interfacial polymerization as an effective synthesis approach for polypyrrole/MWCNTs nanocomposite with impressive nonlinear optical properties. Opt. Mater..

[110] Shi K., Zhitomirsky I. (2013). Polypyrrole nanofiber–carbon nanotube electrodes for supercapacitors with high mass loading obtained using an organic dye as a co-dispersant. J. Mater. Chem. A.

[111] Patil B.H., Shinde S.S., Lokhande C.D. (2013). Synthesis of polypyrrole thin film by SILAR method for supercapacitor application. AIP Conf. Proc..

[112] Thakur A.V., Lokhande B.J. (2019). Effect of the molar concentration of pyrrole monomer on the rate of polymerization, growth and hence the electrochemical behavior of highly pristine PPy flexible electrodes. Heliyon.

[113] Huyen D.N., Tung N.T., Vinh T.D., Thien N.D. (2012). Synergistic Effects in the Gas Sensitivity of Polypyrrole/Single Wall Carbon Nanotube Composites. Sensors.

[114] German N., Ramanaviciene A., Ramanavicius A. (2019). Formation of Polyaniline and Polypyrrole Nanocomposites with Embedded Glucose Oxidase and Gold Nanoparticles. Polymers.

[115] Hashemi Monfared A., Jamshidi M. (2019). Synthesis of polyaniline/titanium dioxide nanocomposite (PAni/TiO_2_) and its application as photocatalyst in acrylic pseudo paint for benzene removal under UV/VIS lights. Prog. Org. Coat..

[116] Salem A.A., Grgur B.N. (2017). Corrosion of Mild Steel with Composite Alkyd Polyanilinebenzoate Coating. Int. J. Electrochem. Sci..

[117] Zhang Y., Xiao R., Liao X., Ma Z., Huang Y., Li Q. (2020). Polyaniline/Copper Composite Anode Current Collectors Prepared through Electrochemical Polymerization for Lithium-Ion Batteries. ChemElectroChem.

[118] Long Y.-Z., Chen Z., Zhang X., Zhang J., Liu Z. (2004). Electrical properties of multi-walled carbon nanotube/polypyrrole nanocables: Percolation-dominated conductivity. J. Phys. D Appl. Phys..

[119] Imani A., Farzi G., Ltaief A. (2013). Facile synthesis and characterization of polypyrrole-multiwalled carbon nanotubes by in situ oxidative polymerization. Int. Nano Lett..

[120] Smitha M.G., Murugendrappa M.V. (2020). Structural, Electrical, Thermal and Transport Properties of Poly Pyrrole/La_0.7_Ca_0.3_MnO_3_ Perovskite Manganite Nano Composite Studies Above Room Temperature. J. Inorg. Organomet. Polym. Mater..

[121] Wu T.-M., Chang H.-L., Lin Y.-W. (2009). Synthesis and characterization of conductive polypyrrole/multi-walled carbon nanotubes composites with improved solubility and conductivity. Compos. Sci. Technol..

[122] Lim T., Oh K.W., Kim S. (2012). Polypyrrole/MWCNT-gr-PSSA Composite for Flexible and Highly Conductive Transparent Film. J. Appl. Polym. Sci..

[123] Milakin K.A., Capáková Z., Acharya U., Vajďák J., Morávková Z., Hodan J., Humpolíček P., Bober P. (2020). Biocompatible and antibacterial gelatin-based polypyrrole cryogels. Polymer.

[124] Li Y., Bober P., Trchová M., Stejskal J. (2017). Polypyrrole prepared in the presence of methyl orange and ethyl orange: Nanotubes versus globules in conductivity enhancement. J. Mater. Chem. C.

[125] Stejskal J., Prokeš J. (2020). Conductivity and morphology of polyaniline and polypyrrole prepared in the presence of organic dyes. Synth. Met..

[126] Karaer Yağmur H. (2020). Synthesis and characterization of conducting polypyrrole/bentonite nanocomposites and in-situ oxidative polymerization of pyrrole: Adsorption of 4-nitrophenol by polypyrrole/bentonite nanocomposite. Chem. Eng. Commun..

[127] Gomes E.C.L., Oliveira M.A.S. (2012). Chemical Polymerization of Aniline in Hydrochloric Acid (HCl) and Formic Acid (HCOOH) Media. Differences Between the Two Synthesized Polyanilines. Am. J. Polym. Sci..

[128] Bahrami A., Talib Z.A., Yunus W.M.M., Behzad K., Abdi M., Din F.U. (2012). Low temperature hall effect investigation of conducting polymer-carbon nanotubes composite network. Int. J. Mol. Sci..

[129] Kaneto K., Yoshino K. (1986). Hall Effect and Reflectance in Conducting Polypyrrole Films. J. Phys. Soc. Jpn..

[130] Xie J., Pan W., Guo Z., Jiao S.S., Ping Yang L. (2019). In situ polymerization of polypyrrole on cotton fabrics as flexible electrothermal materials. J. Eng. Fibers Fabr..

[131] Zhao Y., Tang G.-S., Yu Z.-Z., Qi J.-S. (2012). The effect of graphite oxide on the thermoelectric properties of polyaniline. Carbon.

[132] Zhang A.-Q., Xiao Y.-H., Lu L.-Z., Wang L.-Z., Li F. (2013). Polypyrrole/MnO_2_ composites and their enhanced electrochemical capacitance. J. Appl. Polym. Sci..

[133] Zhang A.-Q., Zhang Y., Wang L.-Z., Li X.-F. (2011). Electrosynthesis and capacitive performance of polyaniline–polypyrrole composite. Polym. Compos..

[134] Daş E., Yurtcan A.B. (2016). Effect of carbon ratio in the polypyrrole/carbon composite catalyst support on PEM fuel cell performance. Int. J. Hydrogen Energy.

[135] Zhang K., Zhang X., Zhao X., Gai X., An W., Fang G., Zhang A., Chen X. (2019). Polypyrrole coated Fe_3_O_4_ nanoparticles decorated carbon nanotubes nanocomposites and the microwave absorption properties. J. Mater. Sci. Mater. Electron..

[136] Petrovski A., Paunović P., Avolio R., Errico M.E., Cocca M., Gentile G., Grozdanov A., Avella M., Barton J., Dimitrov A. (2017). Synthesis and characterization of nanocomposites based on PANI and carbon nanostructures prepared by electropolymerization. Mater. Chem. Phys..

