# Supplementary material for: CVD Conditions for MWCNTs Production and Their Effects on the Optical and Electrical Properties of PPy/MWCNTs, PANI/MWCNTs Nanocomposites by In Situ Electropolymerization
Source: Polymers (Basel). 2021 Jan 22;13(3):351. doi: 10.3390/polym13030351 (PMC7865428; doi:10.3390/polym13030351)
Supplement: Supplementary file 1 [file polymers-13-00351-s001.pdf]

Supplementary material

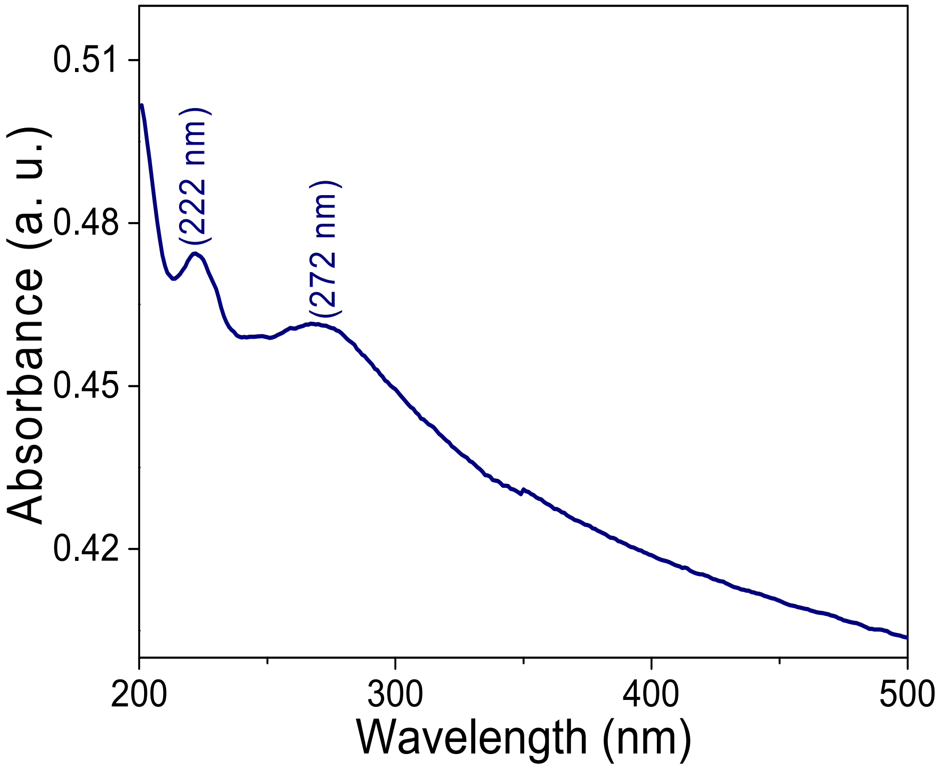

Figure S1. UV-vis spectrum of purified commercial MWCNTs.

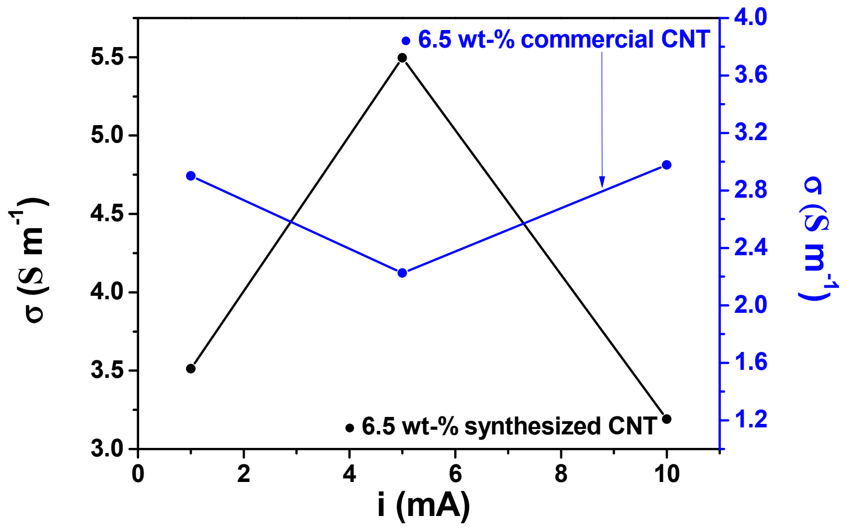

Figure S2. Comparison of electrical conductivity of PANI composites using the synthesized and commercial nanotubes with an amount of 6.5 wt-%.

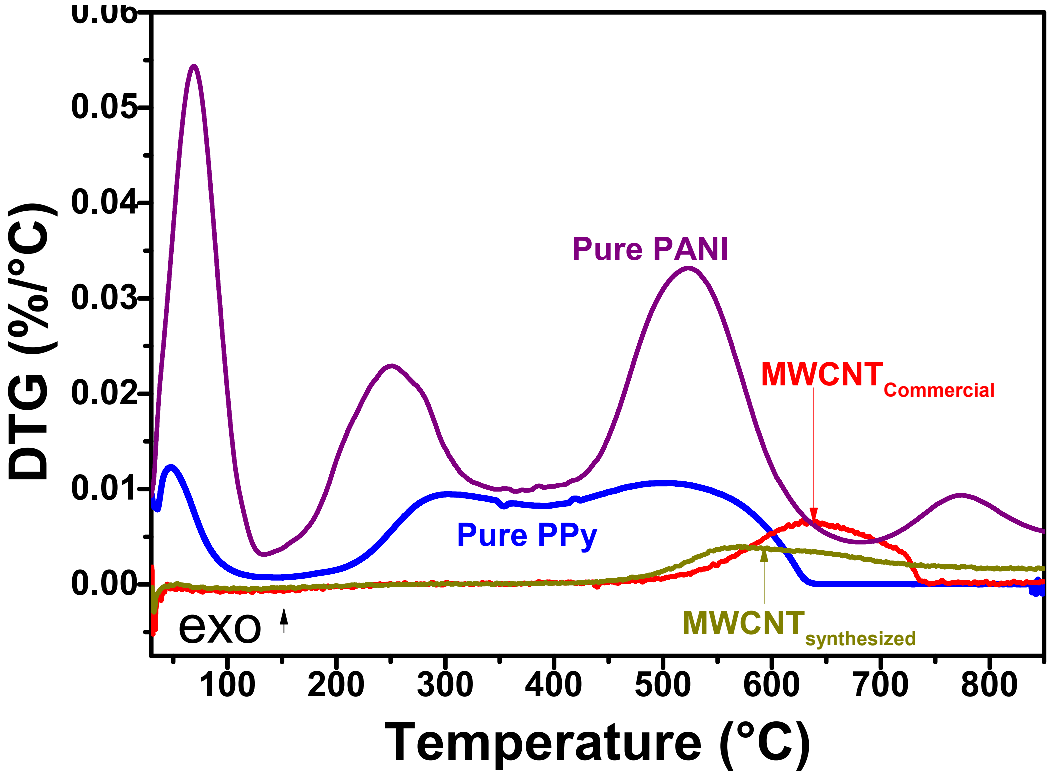

Figure S3. Derivative thermogravimetric analysis of PPy, MW carbon nanotubes synthesized and commercial.

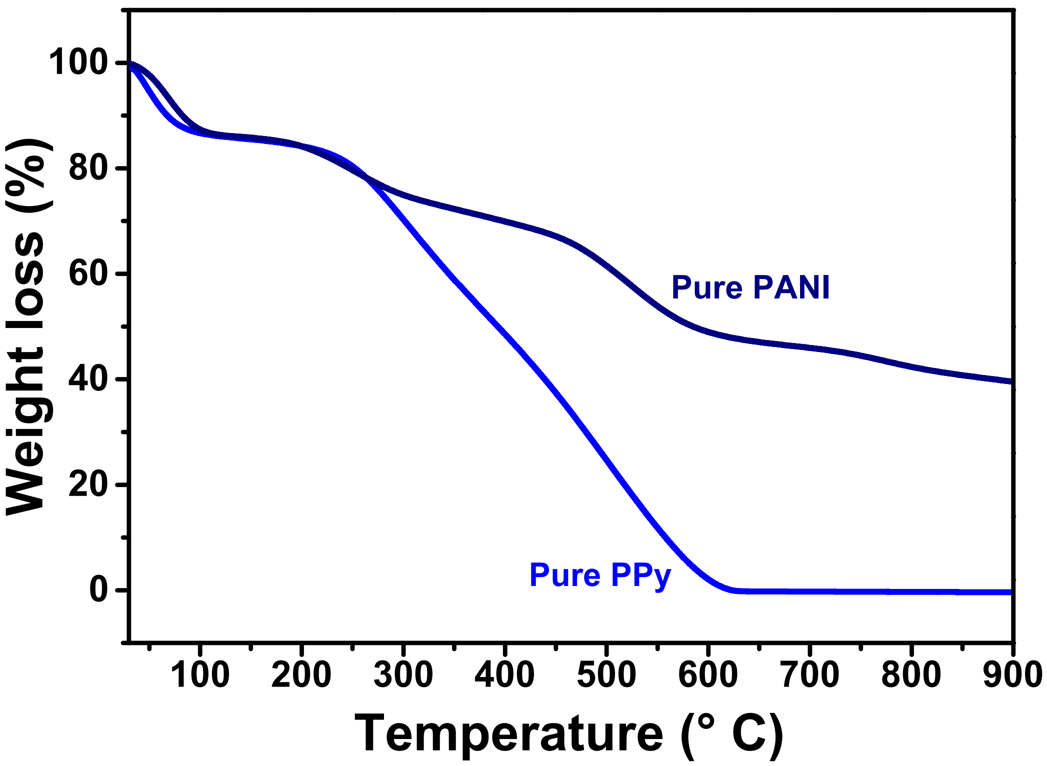

Figure S4. TGA thermograms of pure polymers.
